# Existence and global asymptotic stability criteria for nonlinear neutral-type neural networks involving multiple time delays using a quadratic-integral Lyapunov functional

**DOI:** 10.1186/s13662-021-03274-3

**Published:** 2021-02-17

**Authors:** Yousef Gholami

**Affiliations:** Department of Applied Mathematics, Sahand University of Technology, P.O. Box: 51335-1996, Tabriz, Iran

**Keywords:** 68T07, 34D23, 34K20, 34A34, 34K40, Neural networks, Time-delay, Nonlinearity, Neutrality, Lyapunov functional, Global asymptotic stability

## Abstract

In this paper we consider a standard class of the neural networks and propose an investigation of the global asymptotic stability of these neural systems. The main aim of this investigation is to define a novel Lyapunov functional having quadratic-integral form and use it to reach a stability criterion for the under study neural networks. Since some fundamental characteristics, such as nonlinearity, including time-delays and neutrality, help us design a more realistic and applicable model of neural systems, we will use all of these factors in our neural dynamical systems. At the end, some numerical simulations are presented to illustrate the obtained stability criterion and show the essential role of the time-delays in appearance of the oscillations and stability in the neural networks.

## Introduction

During recent past, it has been shown that wide classes of the real world phenomena can be stated as neural networks. This advantage makes the neural networks powerful resources to study and investigate the mentioned phenomena. On the other hand, unifying various categories of the real life problems in the framework of the neural networks helps us transform these natural phenomena into essentially mathematical engineering problems. So, we can restrict ourselves to investigating the neural networks instead of multi-oriented researches on the aforementioned topics. In this way, the monographs [1, 14] can be helpful. The concept of the neural networks will be more important in theory and in applications if we combine them with time-delays to reach time-delay neural networks (the importance of the time-delay systems can be learned from the monograph [16]). Thanks to the time-delay neural networks, one can study qualitative dynamics of some of the most important bioscience problems, such as the dynamics of diabetes, population dynamics and epidemiology, for instance, time-delay neural networks are capable of making a research field the study of evolutionary dynamics of the COVID-19 pandemic virus. This ability can be summarized in the fact that in time-delay dynamical systems derivatives of unknown functions at particular times can be described in terms of the values of those functions at previous times. In this case, the studied neural networks have great potential to describe rising/falling oscillations, as well as stability/instability in their qualitative dynamics. Let us proceed a bit further. If time-delays appear both in the state of the interconnecting neurons and their derivatives, then the studied dynamical system is said to be a neutral-type time-delay neural network It is expected that these advanced neural networks represent a complete characterization of the neural systems having extended applications in engineering problems. Here, we suggest some of the recent most motivating research papers related to the neutral-type time-delay neural networks and the cited bibliography therein for more consultation on this topic; see [2–8, 11, 24, 25]. The nonlinear nature of the dynamical systems makes them an excellent platform to study the real world phenomena and their engineering refinements, so in the light of the nonlinear neutral-type time-delay neural networks one can concentrate on engineering and artificial intelligence problems such as intelligent recognition processes, including speech recognition, lip reading, handwriting recognition, image recognition, pattern and sequence recognition, data processing, blind signal separation, email spam filtering, signal processing, control problems, fixed point computations, function approximation, optimization problems, and many other real life problems. These applications and corresponding information can be represented in the case that the states of neurons become stable. In other words, as has been proven, such neural systems are required to have constant equilibrium points independent of the initial data that are globally asymptotically stable, see [26]. This is why stabilization of the neural networks has recently attracted wide audience and witnessed day-to-day growing investigations. In this way, the interested follower is advised to study the following papers: [9, 10, 13, 15, 17–23, 26–44]. For convenience let us make a convention here. From now on, we call the nonlinear neutral-type time-delay neural networks as the NNTDNNs.

At the end of this section, we state that the rest of the paper will be organized is follows. In Sect. 2, we first define the main NNTDNNs that will be stabilized later. Also some basic setting and discussions will be made here. Section 3, the main part of the paper, includes the stability analysis to achieve global asymptotic stabilization of the main neural system. Prior to this analysis, by the use of the coincidence degree theory, it will be shown under which conditions the studied NNTDNN has at least one solution to be stabilized. In Sect. 4, we present some numerical simulations to verify the validity of the presented stability criterion. Finally, in Sect. 5, we summarize the solvability and stability criteria presented in this paper.

## Formulation and basic setting

Prior to presenting the stabilization process, we introduce the mathematical model of the dynamical neural system that has described above. As stated, our neural network contains multiple discrete time-delays in the states of the interconnecting neurons and further multiple discrete time-delays in the time derivatives of states of these neurons. Accordingly, our desired NNTDNN is introduced as follows:

2.1 $$ \begin{aligned} \dot{y}_{i}(t):={}&{-}c_{i}y_{i}(t)+ \sum_{j=1}^{n}a_{ij}g_{j} \bigl(y_{j}(t) \bigr)+\sum_{j=1}^{n}b_{ij}g_{j} \bigl(y_{j}(t-\tau _{ij}) \bigr) \\ &{}+ \sum _{j=1}^{n}e_{ij}f_{j} \bigl(\dot{y}_{j}(t-\zeta _{ij}) \bigr)+u_{i},\quad i=1,2,\dots ,n, \end{aligned} $$

with the following properties:

$(P_{1})$
$y_{i}(t)$ denotes the state of the *i*th neuron;
$(P_{2})$
*n* presents the number of the neurons;
$(P_{3})$
$c_{i}$ is some positive real constant;
$(P_{4})$
$a_{ij}$ and $b_{ij}$ are real constants;
$(P_{5})$
$e_{ij}$ is the neutral coefficient;
$(P_{6})$
$\tau _{ij}$ is the time delay of the neuron’s state;
$(P_{7})$
$\zeta _{ij}$ is the neutral time delay;
$(P_{8})$
$g_{j}\in C (\mathbb{R},\mathbb{R} )$ is a nonlinear activation function for which there exists a positive real constant $M_{1}$ such that $|g_{j}(x)|\leq M_{1}$. In addition, $g_{j}$ obeys the Lipschitz-continuity condition, that is, there exists a positive real constant $l_{j}$ such that
  $$ \bigl\vert g_{j}(v)-g_{j}(w) \bigr\vert \leq l_{j} \vert v-w \vert , \quad v,w\in \mathbb{R}, v\neq w, $$
$(P_{9})$
$f_{j}\in C (\mathbb{R},\mathbb{R} )$ is a given function with the property that there exists a positive real positive constant $M_{2}$ such that $|f_{j}(x)|\leq M_{2}$. Furthermore, $f_{j}$ is a Lipschitz-continuous function, so that there exists a positive real constant $m_{j}$ such that
  $$ \bigl\vert f_{j}(v)-f_{j}(w) \bigr\vert \leq m_{j} \vert v-w \vert , \quad v,w\in \mathbb{R}, v\neq w, $$
$(P_{10})$
$u_{i}$ is a constant (an external input);
$(P_{11})$
$\eta :=\max \{\tau _{ij}\mid i,j=1,2,\dots ,n\}$ and $k:=\max \{\zeta _{ij}\mid i,j=1,2,\dots ,n\}$ with $\delta :=\max \{\eta ,k\}$.

At the end of the detailed statement of the neural model (2.1), we note that the accompanying initial data of the neutral-type neural network (2.1) are introduced by

2.2 $$ y_{j}(t)=\phi _{j}(t)\in C \bigl([- \delta ,0],\mathbb{R} \bigr), \qquad \dot{y}_{j}(t)=\psi _{j}(t)\in C \bigl([-\delta ,0],\mathbb{R} \bigr). $$

This is an opportunity to describe the philosophy of the functional space corresponding to the NNTDNN model (2.1). For the stabilization of the NNTDNN, (2.1) is required to be solvable first, that is, the neutral-type neural system (2.1) has to have at least one solution to be stabilized. So, prior to stability analysis, we have to apply an appropriate solvability procedure that gives us a mathematical key to reach at least one solution of this NNTDNN and, consequently, provides a plan to stabilize this solution. Our preferred solvability key is the coincidence degree theory, which, in order to be applicable, must act on a relevant periodic solution space. So, we introduce this periodic functional space as follows:

2.3 $$\begin{aligned}& Y := \bigl\{ y\mid y=(y_{1},y_{2},\dots ,y_{n})\in C \bigl(\mathbb{R}^{n}, \mathbb{R} \bigr), y(t+T)=y(t) \bigr\} , \end{aligned}$$

2.4 $$\begin{aligned}& \Vert y \Vert _{Y} :=\sum_{i=1}^{n} \Vert y_{i} \Vert _{\infty }, \qquad \Vert y_{i} \Vert _{\infty }:= \max_{t[0,T]} \bigl\vert y_{i}(t) \bigr\vert . \end{aligned}$$

We believe that it is necessary to consult more on the neutral-type time delay neural networks, and on the importance of the time delays and stability methods. But at this moment we provide a brief description of the coincidence degree theory. To find the complementary details, we refer the interested followers to [12], Chaps. IV and V). So, let us start as follows.

### Definition 2.1

Let $\mathcal{Y}$ and $\mathcal{Z}$ be real normed spaces. A linear mapping $L:\operatorname{dom} L\subset \mathcal{Y}\rightarrow \mathcal{Z}$ is called a Fredholm mapping provided it satisfies the following conditions:

(i) ker*L* has finite dimension,
(ii) Im*L* is closed and has finite codimension.

Let *L* be a Fredholm mapping. Then its *index* is given by

$$ \operatorname{Ind} L=\dim \ker L-\operatorname{codim} \operatorname{Im} L. $$

Assume that *L* is a Fredholm mapping with zero index and there exist continuous projectors $P:\mathcal{Y}\rightarrow \mathcal{Y}$ and $Q:\mathcal{Z}\rightarrow \mathcal{Z}$ such that

$$ \operatorname{Im} P=\ker L, \qquad \ker Q=\operatorname{Im} L,\qquad \mathcal{Y}= \ker L \oplus \ker P, \qquad \mathcal{Z}=\operatorname{Im} L\oplus \operatorname{Im} Q. $$

So, one may derive that

$$ L|_{\operatorname{dom} L\cap \ker P}: \operatorname{dom} L\cap \ker P \rightarrow \operatorname{Im} L $$

is invertible. Let us denote the inverse by $K_{P}:\operatorname{Im} L\rightarrow \operatorname{dom} L\cap \ker P$. The generalized inverse of *L* denoted by $K_{P,Q}:Z\rightarrow \operatorname{dom} L\cap \ker P$ is defined by $K_{P,Q}=K_{P}(I-Q)$.

If *L* is a Fredholm mapping with zero index, then for every isomorphism $J:\operatorname{Im} Q\rightarrow \ker L$, the mapping $JQ+K_{P,Q}:Z\rightarrow \operatorname{dom} L$ is an isomorphism and, for every $u\in \operatorname{dom} L$,

$$ (JQ+K_{P,Q})^{-1}u=\bigl(L+J^{-1}P\bigr)u. $$

Here, we define the *L*-compact operators that play an important role in the coincidence degree theory.

### Definition 2.2

Let $L:\operatorname{dom} L\subset \mathcal{Y}\rightarrow \mathcal{Z}$ be a Fredholm mapping, *E* be a metric space, and let $N:E\rightarrow \mathcal{Z}$ be a mapping. Then *N* is called *L*-compact on *E* provided that $QN:E\rightarrow \mathcal{Z}$ is continuous and $K_{P,Q}N:E\rightarrow \mathcal{Y}$ is compact on *E*. In addition, we say that *N* is *L*-completely continuous if it is *L*-compact on every bounded $E\subset \mathcal{Y}$.

Now, having all of these preliminaries, we present the main solvability result as follows.

### Theorem 2.3

*Let* $\Omega \subset \mathcal{Y}$ *be open and bounded*, *L* *be a Fredholm mapping with zero index*, *and let* *N* *be* *L*-*compact on* Ω̅. *Furthermore*, *assume that the following conditions are satisfied*:

(i) $Lu\neq \lambda Nu$ *for every* $(u,\lambda )\in ((\operatorname{dom} L\setminus \ker L)\cap \partial \Omega )\times (0,1)$;
(ii) $Nu\notin \operatorname{Im} L$ *for every* $u\in \ker L\cap \partial \Omega $;
(iii) $\deg (JQ N|_{\ker L\cap \partial \Omega },\Omega \cap \ker L,0)\neq 0$ *with* $Q:\mathcal{Y}\rightarrow \mathcal{Y}$ *a continuous projector such that* $\ker Q=\operatorname{Im} L$ *and* $J:\operatorname{Im} Q\rightarrow \ker L$ *is an isomorphism*.

*Then*, *the equation* $Lu=Nu$ *has at least one solution in* $\operatorname{dom} L\cap \overline{\Omega }$.

We finalize this section with a quick overview of the stability tools for the time delay neural networks and complexities of the multiple essentially distinct time delays to stabilize the corresponding neural systems. To this aim, let us consider the forthcoming cases:

$(C_{1})$
If $\tau _{ij}=\tau _{j}$ and $\zeta _{ij}=\zeta _{j}$, for $i,j=1,2,\dots ,n$, then we have the following NNTDNNs:
  2.5 $$ \begin{aligned} \dot{y}_{i}(t):={}&{-}c_{i}y_{i}(t)+ \sum_{j=1}^{n}a_{ij}g_{j} \bigl(y_{j}(t) \bigr)+\sum_{j=1}^{n}b_{ij}g_{j} \bigl(y_{j}(t-\tau _{j}) \bigr) \\ &{}+ \sum _{j=1}^{n}e_{ij}f_{j} \bigl(\dot{y}_{j}(t-\zeta _{j}) \bigr)+u_{i},\quad i=1,2,\dots ,n. \end{aligned} $$
$(C_{2})$
If $\tau _{ij}=\tau $ and $\zeta _{ij}=\zeta $, for $i,j=1,2,\dots ,n$, then we get the following NNTDNNs:
  2.6 $$ \begin{aligned} \dot{y}_{i}(t):={}&{-}c_{i}y_{i}(t)+ \sum_{j=1}^{n}a_{ij}g_{j} \bigl(y_{j}(t) \bigr)+\sum_{j=1}^{n}b_{ij}g_{j} \bigl(y_{j}(t-\tau ) \bigr) \\ &{}+ \sum_{j=1}^{n}e_{ij}f_{j} \bigl(\dot{y}_{j}(t-\zeta ) \bigr)+u_{i},\quad i=1,2,\dots ,n. \end{aligned} $$

The direct consequences of the NNTDNNs (2.5) and (2.6) is that both of these neural networks can be represented in the vector matrix form:

2.7 $$ \dot{y}(t):=-Cy(t)+Ag\bigl(y(t)\bigr)+Bg\bigl(y(t-\tau ) \bigr)+Ef\bigl(\dot{y}(t-\zeta )\bigr)+u, $$

in which

2.8 $$ C:=\operatorname{diag}(c_{i}>0),\qquad \underbrace{A:= (a_{ij} )_{n\times n},\qquad B:= (b_{ij} )_{n\times n}}_{ \text{interconnection matrices}},\qquad E:=A:= (e_{ij} )_{n \times n}, $$

and

2.9 $$ \begin{aligned} &y(t):=\bigl[y_{1}(t),y_{2}(t),\dots ,y_{n}(t)\bigr]^{T}, \\ & g\bigl(y(t)\bigr):= \bigl[g_{1}\bigl(y_{1}(t)\bigr),g_{2} \bigl(y_{2}(t)\bigr), \dots ,g_{n}\bigl(y_{n}(t) \bigr)\bigr]^{T}, \\ & u:=[u_{1},u_{2},\dots ,u_{n}]^{T}. \end{aligned} $$

Furthermore, the vector matrices $g(y(t-\tau ))$ and $f(\dot{y}(t-\zeta ))$ for the two cases $(C_{1})$ and $(C_{2})$ are respectively represented as follows:

$(V_{1})$
  2.10 $$ \begin{aligned} &g\bigl(y(t-\tau )\bigr) := \bigl[g_{1}\bigl(y_{1}(t-\tau _{1}) \bigr),g_{2}\bigl(y_{2}(t- \tau _{2})\bigr), \dots ,g_{n}\bigl(y_{n}(t-\tau _{n})\bigr) \bigr]^{T}, \\ &f\bigl(\dot{y}(t-\zeta )\bigr) :=\bigl[f_{1}\bigl( \dot{y}_{1}(t-\zeta _{1})\bigr),f_{2}\bigl( \dot{y}_{2}(t- \zeta _{2})\bigr),\dots ,f_{n} \bigl(\dot{y}_{n}(t-\zeta _{n})\bigr)\bigr]^{T}, \end{aligned} $$
  and
$(V_{2})$
  2.11 $$ \begin{aligned} &g\bigl(y(t-\tau )\bigr) := \bigl[g_{1}\bigl(y_{1}(t-\tau )\bigr),g_{2} \bigl(y_{2}(t- \tau )\bigr),\dots ,g_{n} \bigl(y_{n}(t-\tau )\bigr)\bigr]^{T}, \\ &f\bigl(\dot{y}(t-\zeta )\bigr) :=\bigl[f_{1}\bigl( \dot{y}_{1}(t-\zeta )\bigr),f_{2}\bigl( \dot{y}_{2}(t- \zeta )\bigr),\dots ,f_{n}\bigl( \dot{y}_{n}(t-\zeta )\bigr)\bigr]^{T}. \end{aligned} $$

Now, comparing the NNTDNN (2.1) with the vector matrix NNTDNNs (2.7)–(2.9), (2.10) and (2.7)–(2.9), (2.11), we come to the conclusion that the NNTDNN (2.1) cannot be represented in the vector matrix form. This is the main complexity of the multiple essentially distinct time delays. In the vector matrix case (2.7), establishing the stability conditions is easier than deriving stability analysis for the NNTDNN (2.1). So, the stabilization techniques such as LMI, that stands for the linear matrix inequality, are not applicable on the NNTDNN (2.1). As instances of this these cases, we suggest the papers [19, 28–30, 36, 40], and the cited bibliography therein for more consultation. In this case, it is reasonable that we are interested in developing the classic mathematical techniques to reach improved stabilization tools such as the novel Lyapunov functionals. Since the stability analysis of the NNTDNNs is not widely investigated in the literature in comparison with the other techniques, this is a good time to make new investigations on neural dynamical systems like the NNTDNN (2.1).

## Stability analysis

As stated in the introduction of the paper, for the stability analysis of the NNTDNN (2.1), we need a criterion that guarantees the existence of at least one solution for the NNTDNN (2.1) to be stabilized. So, we start with the solvability result as follows.

### Theorem 3.1

*Suppose that hypotheses* $(P_{8})$ *and* $(P_{9})$ *are satisfied*. *Then*, *the NNTDNN* (2.1) *has at least one* *T*-*periodic solution*.

### Proof

Our proof strategy is to show that all conditions of Theorem 2.3 are satisfied and, consequently, we conclude that the neural system (2.1) has at least one solution. So, begin with the definition of the basic operators *L* and *N* as follows:

3.1 $$ L:\operatorname{dom} L=\bigl\{ y(t)\mid y(t)\in Y, \dot{y}(t)\in Y \bigr\} \subset Y \longrightarrow Y,\qquad L(y):=\dot{y}, $$

and

3.2 $$ \begin{aligned} & N:Y\longrightarrow Y,\qquad N(y):= \bigl[(N_{1}x) (t),(N_{2}x) (t), \dots ,(N_{n}x) (t)\bigr]^{T}, \\ &(N_{i}x) (t) =-c_{i}y_{i}(t)+\sum _{j=1}^{n}a_{ij}g_{j} \bigl(y_{j}(t) \bigr)+\sum_{j=1}^{n}b_{ij}g_{j} \bigl(y_{j}(t-\tau _{ij}) \bigr) \\ &\hphantom{(N_{i}x) (t) ={}}{}+ \sum _{j=1}^{n}e_{ij}f_{j} \bigl(\dot{y}_{j}(t-\zeta _{ij}) \bigr)+u_{i},\quad i=1,2, \dots ,n \\ &\hphantom{(N_{i}x) (t) } =\Theta _{i} \bigl(t,y(t),y_{1}(t-\tau _{i1}),y_{2}(t-\tau _{i2}), \dots ,y_{n}(t-\tau _{in}), \\ &\hphantom{(N_{i}x) (t) ={} }\dot{y}_{1}(t-\zeta _{i1}),\dot{y}_{2}(t- \zeta _{i2}),\dots , \dot{y}_{n}(t-\zeta _{in}) \bigr). \end{aligned} $$

Also, let us define the projectors $P:Y\longrightarrow \ker L$ and $Q:Y\longrightarrow Y$ as follows:

3.3 $$ Py=Qy:=\frac{1}{T} \int _{0}^{T}y(t)\,dt. $$

So, we get the following:

$$ \ker L:=\mathbb{R}^{n},\qquad \operatorname{Im} L:= \biggl\{ y\Bigm| y\in Y, \int _{0}^{T}y(t)\,dt=0, i=1,2,\dots ,n \biggr\} . $$

Besides,

$$ \operatorname{Im} P=\ker L,\qquad \ker Q=\operatorname{Im} L, $$

that is,

$$ \operatorname{Ind} L:=\dim \ker L-\operatorname{codim} \operatorname{Im} L=n-n=0. $$

So, this proves that *L* is a Fredholm operator of index zero. Next, we show the *L*-compactness of the operator *N*. To this aim, we first define the operator $K_{P}:\operatorname{Im} L\longrightarrow \ker P\cap \operatorname{dom} L$ as

$$ (K_{P}y ) (t):= \bigl[ (K_{P}y )_{1}(t), (K_{P}y )_{2}(t),\dots , (K_{P}y )_{2}(t) \bigr]^{T}, $$

in which

$$ (K_{P}y )_{i}(t):= \int _{0}^{T}y_{i}(t)\,dt- \frac{1}{T} \int _{0}^{T} \int _{0}^{t}y_{i}(s)\,ds,\quad y=(y_{1},y_{2},\dots ,y_{n}) \in \operatorname{Im}L, i=1,2,\dots ,n. $$

Since we do not want to waste the time and space for proving straightforward exercises, so, for a given open and bounded subset Ω of *Y*, we come to the conclusion that both *QN* and $K_{P}(I-Q)N$ are continuous, and $QN (\overline{\Omega } )$ and $K_{P}(I-Q)N (\overline{\Omega } )$ are both relatively compact, that is, the operator *N* is *L*-compact. In accordance with Theorem 2.3, it is time to prove that the condition (i) is satisfied. The plan is as follows. We will show that if $Lu=\lambda Nu$, then $u\in \Omega $, for a given open and bounded subset Ω, that is, we will prove the counterpart of the assumption (i). So, let us begin with

3.4 $$ Ly=\lambda Ny, \quad \lambda \in (0,1), $$

where $y=(y_{1},y_{2},\dots ,y_{n})\in Y$ is an arbitrary solution of the operator equation (3.4). Equivalently, one has

$$\begin{aligned} \dot{y}_{i}(t) :=&\lambda \Theta _{i} \bigl(t,y(t),y_{1}(t-\tau _{i1}),y_{2}(t- \tau _{i2}),\dots ,y_{n}(t-\tau _{in}), \\ & \dot{y}_{1}(t-\zeta _{i1}), \dot{y}_{2}(t- \zeta _{i2}),\dots ,\dot{y}_{n}(t-\zeta _{in}) \bigr),\quad i=1,2, \dots ,n. \end{aligned}$$

Integrating both sides of the recent neural system over the interval $[0,T]$, for $i=1,2,\dots ,n$, yields

3.5 $$ \begin{aligned} y_{i}(T)-y_{i}(0)&=0 \\ &:=\lambda \int _{0}^{T}\Theta _{i} \bigl(t,y(t),y_{1}(t- \tau _{i1}),y_{2}(t- \tau _{i2}),\dots ,y_{n}(t-\tau _{in}), \\ &\quad \dot{y}_{1}(t- \zeta _{i1}),\dot{y}_{2}(t- \zeta _{i2}),\dots ,\dot{y}_{n}(t-\zeta _{in}) \bigr)\,dt. \end{aligned} $$

The left-hand side of the equality (3.5) implies that there exists $s_{i}\in [0,T]$, for $i=1,2,\dots ,n$, such that

3.6 $$ \begin{aligned} &\Theta _{i} \bigl(s_{i},y(s_{i}),y_{1}(s_{i}- \tau _{i1}),y_{2}(s_{i}- \tau _{i2}),\dots ,y_{n}(s_{i}-\tau _{in}), \\ &\quad \dot{y}_{1}(s_{i}-\zeta _{i1}), \dot{y}_{2}(s_{i}-\zeta _{i2}),\dots ,\dot{y}_{n}(s_{i}-\zeta _{in}) \bigr) =0. \end{aligned} $$

Consequently, comparing (3.6) with (3.2), we arrive at the following equality:

3.7 $$ y_{i}(s_{i})=\sum _{j=1}^{n}\frac{a_{ij}}{c_{i}}g_{j} \bigl(y_{j}(s_{i})\bigr)+ \sum _{j=1}^{n}\frac{b_{ij}}{c_{i}}g_{j} \bigl(y_{j}(s_{i}-\tau _{ij})\bigr)+ \sum _{j=1}^{n}\frac{e_{ij}}{c_{i}}f_{j} \bigl(\dot{y}_{j}(s_{i}-\zeta _{ij})\bigr)+ \frac{u_{i}}{c_{i}}. $$

Hence, we get that

$$ \bigl\vert y_{i}(s_{i}) \bigr\vert \leq \sum _{j=1}^{n}\frac{ \vert a_{ij} \vert }{c_{i}} \bigl\vert g_{j}\bigl(y_{j}(s_{i})\bigr) \bigr\vert +\sum_{j=1}^{n} \frac{ \vert b_{ij} \vert }{c_{i}} \bigl\vert g_{j}\bigl(y_{j}(s_{i}- \tau _{ij})\bigr) \bigr\vert +\sum_{j=1}^{n} \frac{ \vert e_{ij} \vert }{c_{i}} \bigl\vert f_{j}\bigl( \dot{y}_{j}(s_{i}- \zeta _{ij})\bigr) \bigr\vert +\frac{ \vert u_{i} \vert }{c_{i}}. $$

Turning to the hypotheses $(P_{8})$ and $(P_{9})$ leads us to the following inequality:

3.8 $$ \bigl\vert y_{i}(s_{i}) \bigr\vert \leq \sum_{j=1}^{n} \biggl\{ \frac{M_{1} ( \vert a_{ij} \vert + \vert b_{ij} \vert )+ M_{2} \vert e_{ij} \vert }{c_{i}} \biggr\} +\frac{ \vert u_{i} \vert }{c_{i}}. $$

In order to reach the desired conclusion, we have to multiply both sides of the operator equation (3.4) and then integrate over the interval $[0,T]$. In this case, it follows that

3.9 $$ \begin{aligned} \int _{0}^{T}\dot{y}_{i}^{2}(t)\,dt={}& \lambda \int _{0}^{T} \Biggl\{ -c_{i}y_{i}(t) \dot{y}_{i}(t)+\sum_{j=1}^{n}a_{ij}g_{j} \bigl(y_{j}(t)\bigr) \dot{y}_{i}(t)+\sum _{j=1}^{n}b_{ij}g_{j} \bigl(y_{j}(t-\tau _{ij})\bigr)\dot{y}_{i}(t) \\ &{} +\sum_{j=1}^{n}e_{ij}f_{j} \bigl(\dot{y}_{j}(t-\zeta _{ij})\bigr) \dot{y}_{i}(t)+u_{i}\dot{y}_{i}(t) \Biggr\} \,dt \\ ={}&\lambda \int _{0}^{T} \Biggl\{ \sum _{j=1}^{n}a_{ij}g_{j} \bigl(y_{j}(t)\bigr) \dot{y}_{i}(t)+\sum _{j=1}^{n}b_{ij}g_{j} \bigl(y_{j}(t-\tau _{ij})\bigr)\dot{y}_{i}(t) \\ &{}+ \sum_{j=1}^{n}e_{ij}f_{j} \bigl(\dot{y}_{j}(t-\zeta _{ij})\bigr) \dot{y}_{i}(t)+u_{i} \dot{y}_{i}(t) \Biggr\} \,dt. \end{aligned} $$

Therefore, we come to the conclusion that

3.10 $$ \begin{aligned} \int _{0}^{T}\dot{y}_{i}^{2}(t)\,dt \leq{}& \int _{0}^{T} \Biggl\vert \sum _{j=1}^{n}a_{ij}g_{j} \bigl(y_{j}(t)\bigr)\dot{y}_{i}(t)+\sum _{j=1}^{n}b_{ij}g_{j} \bigl(y_{j}(t- \tau _{ij})\bigr)\dot{y}_{i}(t) \\ &{}+ \sum_{j=1}^{n}e_{ij}f_{j} \bigl(\dot{y}_{j}(t- \zeta _{ij})\bigr) \dot{y}_{i}(t)+u_{i}\dot{y}_{i}(t) \Biggr\vert \,dt \\ \leq{}& \int _{0}^{T} \Biggl\{ \Biggl\{ \sum _{j=1}^{n} \vert a_{ij} \vert \bigl\vert g_{j}\bigl(y_{j}(t)\bigr) \bigr\vert + \sum_{j=1}^{n} \vert b_{ij} \vert \bigl\vert g_{j}\bigl(y_{j}(t-\tau _{ij})\bigr) \bigr\vert \\ &{}+\sum_{j=1}^{n} \vert e_{ij} \vert \bigl\vert f_{j}\bigl( \dot{y}_{j}(t-\zeta _{ij})\bigr) \bigr\vert + \vert u_{i} \vert \Biggr\} \bigl\vert \dot{y}_{i}(t) \bigr\vert \Biggr\} \,dt \\ \leq{}& \Biggl\{ \sum_{j=1}^{n} \bigl[M_{1}\bigl( \vert a_{ij} \vert + \vert b_{ij} \vert \bigr)+M_{2} \vert e_{ij} \vert \bigr]+ \vert u_{i} \vert \Biggr\} \int _{0}^{T} \bigl\vert \dot{y}_{i}(t) \bigr\vert \,dt. \end{aligned} $$

In continuation, thanks to the inequality

$$ \biggl( \int _{0}^{T} \bigl\vert w(t) \bigr\vert \,dt \biggr)^{2}\leq T \int _{0}^{T} \bigl\vert w(t) \bigr\vert ^{2}\,dt, $$

we have

3.11 $$ \begin{aligned} \biggl( \int _{0}^{T} \bigl\vert \dot{y}_{i}(t) \bigr\vert \,dt \biggr)^{2}&\leq T \int _{0}^{T} \bigl\vert \dot{y}_{i}(t) \bigr\vert ^{2}\,dt \\ &\leq T \Biggl\{ \sum_{j=1}^{n} \bigl[M_{1}\bigl( \vert a_{ij} \vert + \vert b_{ij} \vert \bigr)+M_{2} \vert e_{ij} \vert \bigr]+ \vert u_{i} \vert \Biggr\} \int _{0}^{T} \bigl\vert \dot{y}_{i}(t) \bigr\vert \,dt. \end{aligned} $$

Equivalently, it has shown that

3.12 $$ \int _{0}^{T} \bigl\vert \dot{y}_{i}(t) \bigr\vert \,dt\leq T \Biggl\{ \sum_{j=1}^{n} \bigl[M_{1}\bigl( \vert a_{ij} \vert + \vert b_{ij} \vert \bigr)+M_{2} \vert e_{ij} \vert \bigr]+ \vert u_{i} \vert \Biggr\} . $$

We combine here the inequalities (3.8) and (3.12), and then arrive at the following inequality:

3.13 $$ \bigl\vert y_{i}(t) \bigr\vert \leq \bigl(c_{i}^{-1}+T \bigr) \Biggl\{ \sum _{j=1}^{n} \bigl[M_{1}\bigl( \vert a_{ij} \vert + \vert b_{ij} \vert \bigr)+M_{2} \vert e_{ij} \vert \bigr]+ \vert u_{i} \vert \Biggr\} . $$

Thus,

$$ \Vert y_{i} \Vert _{\infty }=\max_{t\in [0,T]} \bigl\{ \bigl\vert y_{i}(t) \bigr\vert \mid i=1,2,\dots ,n\bigr\} \leq \bigl(c^{-1}+T \bigr) \Biggl\{ \sum_{j=1}^{n} \bigl[M_{1}\bigl( \vert a_{ij} \vert + \vert b_{ij} \vert \bigr)+M_{2} \vert e_{ij} \vert \bigr]+\xi \Biggr\} , $$

in which

$$ \textstyle\begin{cases} c^{-1}:=\max \{ c_{i}^{-1}\mid i=1,2,\dots ,n \} , \\ \xi :=\max \{ \vert u_{i} \vert \mid i=1,2,\dots ,n\}. \end{cases} $$

In this case, we have proved that

3.14 $$ \Vert y \Vert _{Y}=\sum _{i=1}^{n} \Vert y_{i} \Vert _{\infty }\leq \bigl(c^{-1}+T \bigr) \Biggl\{ \sum _{i=1}^{n}\sum_{j=1}^{n} \bigl[M_{1}\bigl( \vert a_{ij} \vert + \vert b_{ij} \vert \bigr)+M_{2} \vert e_{ij} \vert \bigr]+n\xi \Biggr\} . $$

Let $\gamma :=A+\epsilon $, with

$$ \epsilon >1, \qquad A:= \bigl(c^{-1}+T \bigr) \Biggl\{ \sum _{i=1}^{n} \sum_{j=1}^{n} \bigl[M_{1}\bigl( \vert a_{ij} \vert + \vert b_{ij} \vert \bigr)+M_{2} \vert e_{ij} \vert \bigr]+n \xi \Biggr\} . $$

Then, if we define

$$ \Omega :=\bigl\{ y\in Y\mid \Vert y \Vert _{Y}< \gamma \bigr\} , $$

we have proved that, if $Ly=\lambda Ny$, $\lambda \in (0,1)$, then, $y\in \Omega \cap \operatorname{dom} L$. Equivalently, for each $y\in \partial \Omega \cap \operatorname{dom} L$ and $\lambda \in (0,1)$, we have $Ly\neq \lambda Ny$. So, the condition (i) in Theorem 2.3 is satisfied.

Next, we are going to prove the condition (ii) in Theorem 2.3. To this end, suppose $y\in \partial \Omega \cap \ker L$ is a constant vector $y=(y_{1},y_{2},\dots ,y_{n})\in \mathbb{R}^{n}$, with $\|y\|_{Y}=\gamma $. Then, it follows that

$$ \begin{aligned} \Vert QNy \Vert _{Y}&=\sum _{i=1}^{n} \Vert QN_{i}y \Vert _{\infty }=\sum_{i=1}^{n} \biggl\Vert \frac{1}{T} \int _{0}^{T} (N_{i}y ) (t)\,dt \biggr\Vert _{ \infty } \\ &=\sum_{i=1}^{n} \Biggl\Vert \frac{1}{T} \int _{0}^{T} \Biggl\{ -c_{i}y_{i}(t)+ \sum_{j=1}^{n}a_{ij}g_{j} \bigl(y_{j}(t) \bigr) \\ &\quad {}+\sum_{j=1}^{n}b_{ij}g_{j} \bigl(y_{j}(t-\tau _{ij}) \bigr)+\sum _{j=1}^{n}e_{ij}f_{j} \bigl( \dot{y}_{j}(t-\zeta _{ij}) \bigr)+u_{i} \Biggr\} \,dt \Biggr\Vert _{\infty } \\ &=\sum_{i=1}^{n} \Biggl\Vert \frac{1}{T} \int _{0}^{T} \Biggl\{ -c_{i}y_{i}(t)+ \sum_{j=1}^{n} (a_{ij}+b_{ij} )g_{j} (y_{j} )+ \sum_{j=1}^{n}e_{ij}f_{j} (0 )+u_{i} \Biggr\} \,dt \Biggr\Vert _{ \infty } \\ &\geq c\sum_{i=1}^{n} \Vert y_{i} \Vert _{\infty }-\sum_{i=1}^{n} \sum_{j=1}^{n} \bigl\{ \bigl( \vert a_{ij} \vert + \vert b_{ij} \vert \bigr)M_{1}+ \vert e_{ij} \vert M_{2} \bigr\} \\ &\quad {}-n\xi \bigl(c:= \min \{c_{i}\mid i=1,2,\dots ,n\}\bigr) \\ &=c \Biggl\{ \bigl(c^{-1}+T \bigr) \Biggl\{ \sum _{i=1}^{n}\sum_{j=1}^{n} \bigl[M_{1}\bigl( \vert a_{ij} \vert + \vert b_{ij} \vert \bigr)+M_{2} \vert e_{ij} \vert \bigr]+n\xi \Biggr\} + \epsilon \Biggr\} \\ & \quad {}- \Biggl\{ \sum_{i=1}^{n}\sum _{j=1}^{n} \bigl\{ \bigl( \vert a_{ij} \vert + \vert b_{ij} \vert \bigr)M_{1}+ \vert e_{ij} \vert M_{2} \bigr\} +n\xi \Biggr\} . \end{aligned} $$

After some manipulation, we get that for each $y\in \partial \Omega \cap \ker L$,

3.15 $$ \Vert QNy \Vert _{Y}\geq c \Biggl\{ \epsilon +T \Biggl\{ \sum_{i=1}^{n}\sum _{j=1}^{n} \bigl[M_{1}\bigl( \vert a_{ij} \vert + \vert b_{ij} \vert \bigr)+M_{2} \vert e_{ij} \vert \bigr]+n\xi \Biggr\} \Biggr\} >0. $$

The latter inequality proves that $Ny\notin \operatorname{Im} L$, for each $y\in \partial \Omega \cap \ker L$. So, this has proven that the condition (ii) in Theorem 2.3 holds.

Here, we are in such a position to complete the existence analysis by showing that the condition (iii) in Theorem 2.3 is also fulfilled. To this aim, let us define

3.16 $$ H(\lambda ,y):=-\lambda Cy+(1-\lambda )JQNy,\quad (\lambda ,y)\in [0,1] \times \Omega , $$

in which the isomorphism $J:\operatorname{Im} Q\longrightarrow \ker L$ stands for the identity operator and

$$ C:=\operatorname{diag} (c_{1},c_{2},\dots ,c_{n} )\in M_{n \times n}. $$

So, for each $y\in \partial \Omega \cap \ker L$, it follows that

$$ \bigl\Vert H(\lambda ,y) \bigr\Vert _{Y}:=\sum _{i=1}^{n} \bigl\Vert H(\lambda ,y_{i}) \bigr\Vert _{\infty }:= \sum _{i=1}^{n}\max_{t\in [0,T]} \bigl\{ \bigl\vert H\bigl(\lambda ,y_{i}(t)\bigr) \bigr\vert \bigr\} , $$

such that

$$\begin{aligned} H\bigl(\lambda ,y_{i}(t)\bigr) :=&-\lambda c_{i}y_{i}(t) +\frac{(1-\lambda )}{T} \int _{0}^{T} \Biggl\{ -c_{i}y_{i}(t)+ \sum_{j=1}^{n}a_{ij}g_{j} \bigl(y_{j}(t) \bigr) \\ &{}+\sum_{j=1}^{n}b_{ij}g_{j} \bigl(y_{j}(t- \tau _{ij}) \bigr)+\sum _{j=1}^{n}e_{ij}f_{j} \bigl(\dot{y}_{j}(t- \zeta _{ij}) \bigr)+u_{i} \Biggr\} \,dt. \end{aligned}$$

So, we get

$$ \bigl\vert H\bigl(\lambda ,y_{i}(t)\bigr) \bigr\vert \geq c_{i}y_{i}(t)-\frac{1-\lambda }{T} \int _{0}^{T} \Biggl\{ \sum _{j=1}^{n} \bigl\{ \bigl( \vert a_{ij} \vert + \vert b_{ij} \vert \bigr)M_{1}+ \vert e_{ij} \vert M_{2} \bigr\} + \vert u_{i} \vert \Biggr\} \,dt. $$

Therefore, similar to the previous step, one may derive that

$$ \bigl\Vert H(\lambda ,y) \bigr\Vert _{Y}\geq c \Biggl\{ \epsilon +T \Biggl\{ \sum_{i=1}^{n} \sum _{j=1}^{n} \bigl[M_{1}\bigl( \vert a_{ij} \vert + \vert b_{ij} \vert \bigr)+M_{2} \vert e_{ij} \vert \bigr]+n \xi \Biggr\} \Biggr\} >0, $$

that is, for each $y\in \partial \Omega \cap \ker L$, one has $H(\lambda ,y)\neq 0^{T}$. Then, by the use of degree invariance under a homotopy, we arrive at

$$ \begin{aligned} \deg \bigl(JQN|_{\ker L\cap \partial \Omega },\Omega \cap \ker L,0^{T} \bigr)&=\deg \bigl(H(0,\cdot ),\Omega \cap \ker L,0^{T}\bigr) \\ &=\deg \bigl(H(1,\cdot ),\Omega \cap \ker L,0^{T}\bigr) \\ &=\deg \bigl(-Cy,\Omega \cap \ker L,0^{T} \bigr)\neq 0. \end{aligned} $$

So, the condition (iii) in Theorem 2.3 is fulfilled. Since all conditions of Theorem 2.3 are satisfied, we come to the conclusion that the NNTDNN (2.1) has at least one solution. This completes the proof. □

This situation is at the borderline between the solvability and stability of the NNTDNN (2.1), meaning that it just remains to handle the uniqueness of the existing solution (equilibrium point in view of the dynamical systems) of the NNTDNN (2.1). To this aim, wee just need a little bit of creativity to introduce the transformation $z(t):=y(t)-y^{*}$ where $y^{*}=(y_{1}^{*},y_{2}^{*},\dots ,y_{n}^{*})^{T}\in \mathbb{R}^{n}$ stands for a given equilibrium point of the NNTDNN (2.1). In this case, the NNTDNN (2.1) can be restated as the following transformed NNTDNN:

3.17 $$ \begin{aligned} \dot{z}_{i}(t):={}&{-}c_{i}z_{i}(t)+ \sum_{j=1}^{n}a_{ij} \mathcal{G}_{j} \bigl(z_{j}(t) \bigr)+\sum _{j=1}^{n}b_{ij}\mathcal{G}_{j} \bigl(z_{j}(t- \tau _{ij}) \bigr) \\ &{}+\sum _{j=1}^{n}e_{ij}\mathcal{F}_{j} \bigl(\dot{z}_{j}(t- \zeta _{ij}) \bigr), \quad i=1,2,\dots ,n. \end{aligned} $$

The golden point of the NNTDNN (3.17) is that this neural network has the origin as its unique equilibrium point. This property enables us now to manage the claimed global asymptotic stability analysis. Prior to presenting the stability analysis, we point out that in the new NNTDNN (3.17), all properties of the NNTDNN (2.1) hold, just in the properties $(P_{8})$ and $(P_{9})$ the Lipschitz-continuities will be transformed into the following ones. So, the NNTDNN (3.17) has the following properties:

$(P_{\text{new}})$
Comparing both of the neural networks (2.1) and (3.17), we have
  3.18 $$ (P_{i,\text{new}})=(P_{i}),\quad i=1,2,\dots ,7,10,11; $$
$(P_{8, \text{new}})$
  3.19 $$ \textstyle\begin{cases} \vert \mathcal{G}_{i}(z_{i}(t)) \vert \leq l_{i} \vert z_{i}(t) \vert , \quad i=1,2,\dots ,n, \\ \mathcal{G}_{i}(z_{i}(t)):=g_{i}(z_{i}+y_{i}^{*})-g_{i}(y_{i}^{*}),\quad \text{with } \mathcal{G}_{i}(0)=0, i=1,2,\dots ,n. \end{cases} $$

Furthermore,

$(P_{9, \text{new}})$
  3.20 $$ \textstyle\begin{cases} |\mathcal{F}_{i}(z_{i}(t))|\leq m_{i} \vert z_{i}(t) \vert ,\quad i=1,2,\dots ,n, \\ \mathcal{F}_{i}(z_{i}(t)):=f_{i}(z_{i}+y_{i}^{*})-f_{i}(y_{i}^{*}),\quad \text{with } \mathcal{F}_{i}(0)=0, i=1,2,\dots ,n. \end{cases} $$

Now, we are ready to state and prove the main stability result as the following theorem.

### Theorem 3.2

*Suppose the nonlinear neutral*-*type time*-*delay neural network* (3.17) *satisfies the properties* $(P_{1, \mathrm{new}})$*–*$(P_{11, \mathrm{new}})$. *If* *α* *is a positive constant such that* $0<\alpha <1$, *then the origin of the NNTDNN* (3.17) *is globally asymptotically stable provided that the following conditions are satisfied for each* $i=1,2,\dots ,n$:

3.21 $$\begin{aligned}& \begin{aligned} \epsilon _{1,i}:={}&\alpha \bigl(c_{i}^{2}+ \delta l_{i}^{2} \bigr)+ \frac{1-\alpha }{2}\sum _{j=1}^{n}\bigl\{ \vert a_{ij} \vert + \vert b_{ij} \vert + \vert e_{ij} \vert +l_{i}^{2} \vert a_{ji} \vert \bigr\} \\ &{}-l_{i}^{2}\sum _{j=1}^{n}\sum _{k=1}^{n}\bigl\{ \vert a_{ji} \vert \vert b_{jk} \vert + \vert a_{ji} \vert \vert c_{jk} \vert + \vert a_{ki} \vert \vert a_{kj} \vert \bigr\} >0, \end{aligned} \end{aligned}$$

3.22 $$\begin{aligned}& \epsilon _{2,i}=-c_{i}\frac{1-\alpha }{2}\sum _{j=1}^{n} \vert b_{ji} \vert + \sum_{j=1}^{n}\sum _{k=1}^{n}\bigl\{ \vert a_{jk} \vert \vert b_{ji} \vert + \vert b_{jk} \vert \vert b_{ji} \vert + \vert e_{jk} \vert \vert c_{ji} \vert \bigr\} >0, \end{aligned}$$

3.23 $$\begin{aligned}& \epsilon _{3,i}:=-c_{i}\frac{1-\alpha }{2}\sum _{j=1}^{n} \vert e_{ji} \vert + \sum_{j=1}^{n}\sum _{k=1}^{n}\bigl\{ \vert a_{jk} \vert \vert e_{ji} \vert + \vert b_{jk} \vert \vert e_{ji} \vert + \vert e_{jk} \vert \vert e_{ji} \vert \bigr\} >0. \end{aligned}$$

### Proof

The main strategy to prove this theorem is to define the following quadratic-integral Lyapunov functional:

3.24 $$\begin{aligned}& V\bigl(z(t),\dot{z}(t),t\bigr) :=V_{1}\bigl(z(t),\dot{z}(t),t \bigr)+V_{2}\bigl(z(t),\dot{z}(t),t\bigr), \end{aligned}$$

3.25 $$\begin{aligned}& V_{1}\bigl(z(t),\dot{z}(t),t\bigr) :=\frac{(1+\alpha )}{2}\sum _{i=1}^{n}c_{i}m_{i}^{2}z_{i}^{2}(t)+ \frac{1}{n}\sum_{i=1}^{n}\sum _{j=1}^{n} \int _{t-\zeta _{ij}}^{t} \mathcal{F}_{j}^{2} \bigl(\dot{z}_{j}(s)\bigr)\,ds, \end{aligned}$$

3.26 $$\begin{aligned}& V_{2}\bigl(z(t),\dot{z}(t),t\bigr) :=\delta \sum _{i=1}^{n}\sum_{j=1}^{n} \int _{t- \tau _{ji}}^{t}\mathcal{G}_{j}^{2} \bigl(z_{j}(s)\bigr)\,ds. \end{aligned}$$

In this case, one has

3.27 $$ \begin{aligned} \dot{V}_{1}\bigl(z(t),\dot{z}(t),t\bigr):={}&(1+ \alpha )\sum_{i=1}^{n}c_{i}m_{i}^{2}z_{i}(t) \dot{z}_{i}(t)+\frac{1}{n}\sum _{i=1}^{n}\sum_{j=1}^{n} \mathcal{F}_{j}^{2}\bigl( \dot{z}_{j}(t) \bigr) \\ &{}-\frac{1}{n}\sum_{i=1}^{n} \sum_{j=1}^{n}\mathcal{F}_{j}^{2} \bigl( \dot{z}_{j}(t-\zeta _{ij})\bigr) \end{aligned} $$

and

3.28 $$ \dot{V}_{2}\bigl(z(t),\dot{z}(t),t\bigr):=\delta \sum_{i=1}^{n}\sum _{j=1}^{n} \mathcal{G}_{j}^{2} \bigl(\dot{z}_{j}(t)\bigr)-\delta \sum_{i=1}^{n} \sum_{j=1}^{n} \mathcal{G}_{j}^{2} \bigl(\dot{z}_{j}(t-\tau _{ij})\bigr). $$

We continue keeping in the mind the following key point:

3.29 $$ \frac{1}{n}\sum_{i=1}^{n} \sum_{j=1}^{n}\mathcal{F}_{j}^{2} \bigl(\dot{z}_{j}(t)\bigr)= \sum_{j=1}^{n} \mathcal{F}_{j}^{2}\bigl(\dot{z}_{j}(t) \bigr)=\sum_{i=1}^{n} \mathcal{F}_{i}^{2}\bigl(\dot{z}_{i}(t) \bigr). $$

Thanks to this key point, and concentrating on the first two parts of $\dot{V}_{1}$, given by (3.27), it follows that

3.30 $$\begin{aligned} &(1+\alpha )\sum_{i=1}^{n}c_{i}m_{i}^{2}z_{i}(t) \dot{z}_{i}(t)+ \frac{1}{n}\sum _{i=1}^{n}\sum_{j=1}^{n} \mathcal{F}_{j}^{2}\bigl(\dot{z}_{j}(t) \bigr) \\ &\quad =(1+\alpha )\sum_{i=1}^{n}c_{i}m_{i}^{2}z_{i}(t) \dot{z}_{i}(t)+\sum_{i=1}^{n} \mathcal{F}_{i}^{2}\bigl(\dot{z}_{i}(t) \bigr) \\ &\quad \leq (1+\alpha )\sum_{i=1}^{n}c_{i}m_{i}^{2}z_{i}(t) \dot{z}_{i}(t)+ \sum_{i=1}^{n}m_{i}^{2} \dot{z}_{i}^{2}(t) \quad (\text{the property $(P_{9, \text{new}})$}) \\ &\quad =\sum_{i=1}^{n}m_{i}^{2} \bigl\{ \alpha c_{i}z_{i}(t)+\bigl( \dot{z}_{i}(t)+c_{i}z_{i}(t)\bigr) \bigr\} \dot{z}_{i}(t) \\ &\quad =\sum_{i=1}^{n}m_{i}^{2} \Biggl\{ \alpha c_{i}z_{i}(t)+ \sum _{j=1}^{n}a_{ij} \mathcal{G}_{j} \bigl(z_{j}(t) \bigr)+ \sum_{j=1}^{n}b_{ij} \mathcal{G}_{j} \bigl(z_{j}(t-\tau _{ij}) \bigr)+ \sum_{j=1}^{n}e_{ij} \mathcal{F}_{j} \bigl(\dot{z}_{j}(t-\zeta _{ij}) \bigr) \Biggr\} \dot{z}_{i}(t) \\ &\quad =-\alpha \sum_{i=1}^{n}m_{i}^{2}c_{i}^{2}z_{i}^{2}(t) \end{aligned}$$

3.31 $$\begin{aligned} &\qquad {}+(\alpha -1)\sum_{i=1}^{n}m_{i}^{2}c_{i}z_{i}(t) \sum_{j=1}^{n}a_{ij} \mathcal{G}_{j} \bigl(z_{j}(t) \bigr) \end{aligned}$$

3.32 $$\begin{aligned} &\qquad {}+(\alpha -1)\sum_{i=1}^{n}m_{i}^{2}c_{i}z_{i}(t) \sum_{j=1}^{n}b_{ij} \mathcal{G}_{j} \bigl(z_{j}(t-\tau _{ij}) \bigr) \end{aligned}$$

3.33 $$\begin{aligned} &\qquad {}+(\alpha -1)\sum_{i=1}^{n}m_{i}^{2}c_{i}z_{i}(t) \sum_{j=1}^{n}e_{ij} \mathcal{F}_{j} \bigl(\dot{z}_{j}(t-\zeta _{ij}) \bigr) \end{aligned}$$

3.34 $$\begin{aligned} &\qquad {}+2\sum_{i=1}^{n}m_{i}^{2} \Biggl(\sum_{j=1}^{n}a_{ij} \mathcal{G}_{j} \bigl(z_{j}(t) \bigr) \Biggr) \Biggl( \sum_{j=1}^{n}b_{ij} \mathcal{G}_{j} \bigl(z_{j}(t-\tau _{ij}) \bigr) \Biggr) \end{aligned}$$

3.35 $$\begin{aligned} &\qquad {}+2\sum_{i=1}^{n}m_{i}^{2} \Biggl(\sum_{j=1}^{n}a_{ij} \mathcal{G}_{j} \bigl(z_{j}(t) \bigr) \Biggr) \Biggl( \sum_{j=1}^{n}e_{ij} \mathcal{F}_{j} \bigl(\dot{z}_{j}(t-\zeta _{ij}) \bigr) \Biggr) \end{aligned}$$

3.36 $$\begin{aligned} &\qquad {}+2\sum_{i=1}^{n}m_{i}^{2} \Biggl(\sum_{j=1}^{n}b_{ij} \mathcal{G}_{j} \bigl(z_{j}(t-\tau _{ij}) \bigr) \Biggr) \Biggl(\sum_{j=1}^{n}e_{ij} \mathcal{F}_{j} \bigl(\dot{z}_{j}(t-\zeta _{ij}) \bigr) \Biggr) \end{aligned}$$

3.37 $$\begin{aligned} &\qquad {}+\sum_{i=1}^{n}m_{i}^{2} \Biggl(\sum_{j=1}^{n}a_{ij} \mathcal{G}_{j} \bigl(z_{j}(t) \bigr) \Biggr) \Biggl( \sum_{j=1}^{n}a_{ij} \mathcal{G}_{j} \bigl(z_{j}(t) \bigr) \Biggr) \end{aligned}$$

3.38 $$\begin{aligned} &\qquad {}+\sum_{i=1}^{n}m_{i}^{2} \Biggl(\sum_{j=1}^{n}b_{ij} \mathcal{G}_{j} \bigl(z_{j}(t-\tau _{ij}) \bigr) \Biggr) \Biggl(\sum_{j=1}^{n}b_{ij} \mathcal{G}_{j} \bigl(z_{j}(t-\tau _{ij}) \bigr) \Biggr) \end{aligned}$$

3.39 $$\begin{aligned} &\qquad {}+\sum_{i=1}^{n}m_{i}^{2} \Biggl(\sum_{j=1}^{n}e_{ij} \mathcal{F}_{j} \bigl(\dot{z}_{j}(t-\zeta _{ij}) \bigr) \Biggr) \Biggl(\sum_{j=1}^{n}e_{ij} \mathcal{F}_{j} \bigl(\dot{z}_{j}(t-\zeta _{ij}) \bigr) \Biggr). \end{aligned}$$

In continuation, we are going to bound the multiple series (3.31)–(3.39) one-by-one as follows:

$$\begin{aligned}& (\alpha -1)\sum_{i=1}^{n}m_{i}^{2}c_{i}z_{i}(t) \sum_{j=1}^{n}a_{ij} \mathcal{G}_{j} \bigl(z_{j}(t) \bigr) \\ & \quad = (\alpha -1)\sum_{i=1}^{n}\sum _{j=1}^{n}m_{i}^{2}c_{i}a_{ij}z_{i}(t) \mathcal{G}_{j} \bigl(z_{j}(t) \bigr) \\ & \quad \leq (1-\alpha )\sum_{i=1}^{n}\sum _{j=1}^{n}m_{i}^{2}c_{i} \vert a_{ij} \vert \bigl\vert z_{i}(t) \bigr\vert \bigl\vert \mathcal{G}_{j} \bigl(z_{j}(t) \bigr) \bigr\vert \\ & \quad \leq \frac{1-\alpha }{2}\sum_{i=1}^{n} \sum_{j=1}^{n}m_{i}^{2}c_{i} \vert a_{ij} \vert z_{i}^{2}(t)+ \frac{1-\alpha }{2}\sum_{i=1}^{n}\sum _{j=1}^{n}m_{i}^{2}c_{i} \vert a_{ij} \vert \mathcal{G}_{j}^{2} \bigl(z_{j}(t) \bigr) \\ & \quad \leq \frac{1-\alpha }{2}\sum_{i=1}^{n} \sum_{j=1}^{n}m_{i}^{2}c_{i} \vert a_{ij} \vert z_{i}^{2}(t)+ \frac{1-\alpha }{2}\sum_{i=1}^{n}\sum _{j=1}^{n}m_{i}^{2}c_{i} \vert a_{ij} \vert l_{j}^{2}z_{j}^{2}(t) \\ & \qquad (\text{according to the property $(P_{8, \text{new}})$}) \\& \quad = \frac{1-\alpha }{2}\sum_{i=1}^{n} \sum_{j=1}^{n}m_{i}^{2}c_{i} \vert a_{ij} \vert z_{i}^{2}(t)+ \frac{1-\alpha }{2}\sum_{i=1}^{n}\sum _{j=1}^{n}m_{i}^{2}c_{i} \vert a_{ji} \vert l_{i}^{2}z_{i}^{2}(t). \end{aligned}$$

Therefore, we get

3.40 $$ (\alpha -1)\sum_{i=1}^{n}m_{i}^{2}c_{i}z_{i}(t) \sum_{j=1}^{n}a_{ij} \mathcal{G}_{j} \bigl(z_{j}(t) \bigr)\leq \frac{1-\alpha }{2}\sum_{i=1}^{n} \sum _{j=1}^{n}m_{i}^{2}c_{i} \bigl( \vert a_{ij} \vert +l_{i}^{2} \vert a_{ji} \vert \bigr)z_{i}^{2}(t). $$

Let us now consider (3.32). Then,

$$\begin{aligned}& (\alpha -1)\sum_{i=1}^{n}m_{i}^{2}c_{i}z_{i}(t) \sum_{j=1}^{n}b_{ij} \mathcal{G}_{j} \bigl(z_{j}(t-\tau _{ij}) \bigr) \\& \quad = (\alpha -1)\sum_{i=1}^{n}\sum _{j=1}^{n}m_{i}^{2}c_{i}b_{ij}z_{i}(t) \mathcal{G}_{j} \bigl(z_{j}(t-\tau _{ij}) \bigr) \\& \quad \leq (1-\alpha )\sum_{i=1}^{n}\sum _{j=1}^{n}m_{i}^{2}c_{i} \vert b_{ij} \vert \bigl\vert z_{i}(t) \bigr\vert \bigl\vert \mathcal{G}_{j} \bigl(z_{j}(t-\tau _{ij}) \bigr) \bigr\vert \\& \quad \leq \frac{1-\alpha }{2}\sum_{i=1}^{n} \sum_{j=1}^{n}m_{i}^{2}c_{i} \vert b_{ij} \vert z_{i}^{2}(t)+ \frac{1-\alpha }{2}\sum_{i=1}^{n}\sum _{j=1}^{n}m_{i}^{2}c_{i} \vert b_{ij} \vert \mathcal{G}_{j}^{2} \bigl(z_{j}(t-\tau _{ij}) \bigr) \\& \quad \leq \frac{1-\alpha }{2}\sum_{i=1}^{n} \sum_{j=1}^{n}m_{i}^{2}c_{i} \vert b_{ij} \vert z_{i}^{2}(t)+ \frac{1-\alpha }{2}\sum_{i=1}^{n}\sum _{j=1}^{n}m_{i}^{2}c_{i} \vert b_{ij} \vert l_{j}^{2}z_{j}^{2}(t- \tau _{ij}) \\& \qquad (\text{according to the property $(P_{8, \text{new}})$}) \\& \quad = \frac{1-\alpha }{2}\sum_{i=1}^{n} \sum_{j=1}^{n}m_{i}^{2}c_{i} \vert b_{ij} \vert z_{i}^{2}(t)+ \frac{1-\alpha }{2}\sum_{i=1}^{n}\sum _{j=1}^{n}m_{i}^{2}c_{i} \vert b_{ji} \vert l_{i}^{2}z_{i}^{2}(t- \tau _{ji}), \end{aligned}$$

that is,

3.41 $$ \begin{aligned} &(\alpha -1)\sum_{i=1}^{n}m_{i}^{2}c_{i}z_{i}(t) \sum_{j=1}^{n}a_{ij} \mathcal{G}_{j} \bigl(z_{j}(t-\tau _{ij}) \bigr) \\ &\quad \leq \frac{1-\alpha }{2}\sum_{i=1}^{n} \sum_{j=1}^{n}m_{i}^{2}c_{i} \vert b_{ij} \vert z_{i}^{2}(t)+ \frac{1-\alpha }{2}\sum_{i=1}^{n}\sum _{j=1}^{n}m_{i}^{2}c_{i} \vert b_{ji} \vert l_{i}^{2}z_{i}^{2}(t- \tau _{ji}). \end{aligned} $$

At this step, if we consider (3.33) and arrive at

$$\begin{aligned}& (\alpha -1)\sum_{i=1}^{n}m_{i}^{2}c_{i}z_{i}(t) \sum_{j=1}^{n}e_{ij} \mathcal{F}_{j} \bigl(\dot{z}_{j}(t-\zeta _{ij}) \bigr) \\& \quad = (\alpha -1)\sum_{i=1}^{n}\sum _{j=1}^{n}m_{i}^{2}c_{i}e_{ij}z_{i}(t) \mathcal{F}_{j} \bigl(\dot{z}_{j}(t-\zeta _{ij}) \bigr) \\& \quad \leq (1-\alpha )\sum_{i=1}^{n}\sum _{j=1}^{n}m_{i}^{2}c_{i} \vert e_{ij} \vert \bigl\vert z_{i}(t) \bigr\vert \bigl\vert \mathcal{F}_{j} \bigl(\dot{z}_{j}(t- \zeta _{ij}) \bigr) \bigr\vert \\& \quad \leq \frac{1-\alpha }{2}\sum_{i=1}^{n} \sum_{j=1}^{n}m_{i}^{2}c_{i} \vert e_{ij} \vert z_{i}^{2}(t)+ \frac{1-\alpha }{2}\sum_{i=1}^{n}\sum _{j=1}^{n}m_{i}^{2}c_{i} \vert e_{ij} \vert \mathcal{F}_{j}^{2} \bigl(\dot{z}_{j}(t-\zeta _{ij}) \bigr) \\& \quad \leq \frac{1-\alpha }{2}\sum_{i=1}^{n} \sum_{j=1}^{n}m_{i}^{2}c_{i} \vert e_{ij} \vert z_{i}^{2}(t)+ \frac{1-\alpha }{2}\sum_{i=1}^{n}\sum _{j=1}^{n}m_{i}^{2}c_{i} \vert e_{ij} \vert m_{j}^{2}z_{j}^{2}(t- \zeta _{ij}) \\& \qquad (\text{according to the property $(P_{9, \text{new}})$}) \\& \quad = \frac{1-\alpha }{2}\sum_{i=1}^{n} \sum_{j=1}^{n}m_{i}^{2}c_{i} \vert e_{ij} \vert z_{i}^{2}(t)+ \frac{1-\alpha }{2}\sum_{i=1}^{n}\sum _{j=1}^{n}m_{i}^{4}c_{i} \vert e_{ji} \vert \dot{z}_{i}^{2}(t- \zeta _{ji}). \end{aligned}$$

Equivalently,

3.42 $$ \begin{aligned} &(\alpha -1)\sum_{i=1}^{n}m_{i}^{2}c_{i}z_{i}(t) \sum_{j=1}^{n}e_{ij} \mathcal{F}_{j} \bigl(\dot{z}_{j}(t-\zeta _{ij}) \bigr) \\ &\quad \leq \frac{1-\alpha }{2}\sum _{i=1}^{n}\sum_{j=1}^{n}m_{i}^{2}c_{i} \vert e_{ij} \vert z_{i}^{2}(t)+ \frac{1-\alpha }{2}\sum_{i=1}^{n}\sum _{j=1}^{n}m_{i}^{4}c_{i} \vert e_{ji} \vert \dot{z}_{i}^{2}(t- \zeta _{ji}). \end{aligned} $$

Now, it is time to estimate the triple group (3.34)–(3.36). So, we begin with (3.34) as follows:

$$\begin{aligned}& 2\sum_{i=1}^{n}m_{i}^{2} \sum_{j=1}^{n}a_{ij} \mathcal{G}_{j}\bigl(z_{j}(t)\bigr)\sum _{j=1}^{n}b_{ij}\mathcal{G}_{j} \bigl(z_{j}(t- \tau _{ij})\bigr) \\& \quad = 2\sum_{i=1}^{n}m_{i}^{2} \sum_{j=1}^{n}a_{ij} \mathcal{G}_{j}\bigl(z_{j}(t)\bigr) \sum _{k=1}^{n}b_{ik}\mathcal{G}_{k} \bigl(z_{k}(t-\tau _{ik})\bigr) \\& \quad = 2\sum_{i=1}^{n}\sum _{j=1}^{n}\sum_{k=1}^{n}m_{i}^{2}a_{ij}b_{ik} \mathcal{G}_{j}\bigl(z_{j}(t)\bigr) \mathcal{G}_{k}\bigl(z_{k}(t-\tau _{ik}) \bigr) \\& \quad \leq 2\sum_{i=1}^{n}\sum _{j=1}^{n}\sum_{k=1}^{n}m_{i}^{2} \vert a_{ij} \vert \vert b_{ik} \vert \bigl\vert \mathcal{G}_{j}\bigl(z_{j}(t)\bigr) \bigr\vert \bigl\vert \mathcal{G}_{k}\bigl(z_{k}(t-\tau _{ik})\bigr) \bigr\vert \\& \quad \leq \sum_{i=1}^{n}\sum _{j=1}^{n}\sum_{k=1}^{n}m_{i}^{2} \vert a_{ij} \vert |b_{ik}| \bigl\vert \mathcal{G}_{j}^{2}\bigl(z_{j}(t)\bigr) \bigr\vert +\sum_{i=1}^{n}\sum _{j=1}^{n}\sum_{k=1}^{n}m_{i}^{2}|a_{ij}||b_{ik}| \mathcal{G}_{k}^{2}\bigl(z_{k}(t-\tau _{ik})\bigr) \\& \quad = \sum_{i=1}^{n}\sum _{j=1}^{n}\sum_{k=1}^{n}m_{i}^{2} \vert a_{ji} \vert |b_{jk}| \bigl\vert \mathcal{G}_{i}^{2}\bigl(z_{i}(t)\bigr) \bigr\vert +\sum_{i=1}^{n}\sum _{j=1}^{n}\sum_{k=1}^{n}m_{i}^{2}|a_{jk}||b_{ki}| \mathcal{G}_{i}^{2}\bigl(z_{i}(t-\tau _{ki})\bigr) \\& \quad \leq \sum_{i=1}^{n}\sum _{j=1}^{n}\sum_{k=1}^{n}m_{i}^{2} \vert a_{ji} \vert \vert b_{jk} \vert l_{i}^{2}z_{i}^{2}(t)+ \sum _{i=1}^{n}\sum _{j=1}^{n}\sum_{k=1}^{n}m_{i}^{2} \vert a_{jk} \vert \vert b_{ki} \vert l_{i}^{2}z_{i}^{2}(t- \tau _{ki}) \\& \qquad (\text{according to the property $(P_{8, \text{new}})$}) \\& \quad = \sum_{i=1}^{n}\sum _{j=1}^{n}\sum_{k=1}^{n}m_{i}^{2} \vert a_{ji} \vert \vert b_{jk} \vert l_{i}^{2}z_{i}^{2}(t)+ \sum _{i=1}^{n}\sum _{j=1}^{n}\sum_{k=1}^{n}m_{i}^{2} \vert a_{jk} \vert \vert b_{ji} \vert l_{i}^{2}z_{i}^{2}(t- \tau _{ji}). \end{aligned}$$

Hence, we have shown that

3.43 $$ \begin{aligned} &2\sum_{i=1}^{n}m_{i}^{2} \sum_{j=1}^{n}a_{ij} \mathcal{G}_{j}\bigl(z_{j}(t)\bigr) \sum _{j=1}^{n}b_{ij}\mathcal{G}_{j} \bigl(z_{j}(t-\tau _{ij})\bigr) \\ &\quad \leq \sum _{i=1}^{n} \sum_{j=1}^{n} \sum_{k=1}^{n}m_{i}^{2} \vert a_{ji} \vert \vert b_{jk} \vert l_{i}^{2}z_{i}^{2}(t)+ \sum _{i=1}^{n}\sum _{j=1}^{n}\sum_{k=1}^{n}m_{i}^{2} \vert a_{jk} \vert \vert b_{ji} \vert l_{i}^{2}z_{i}^{2}(t- \tau _{ji}). \end{aligned} $$

Here we take a look at (3.35). In this case,

$$\begin{aligned}& 2\sum_{i=1}^{n}m_{i}^{2} \sum_{j=1}^{n}a_{ij} \mathcal{G}_{j}\bigl(z_{j}(t)\bigr)\sum _{j=1}^{n}e_{ij}\mathcal{F}_{j} \bigl(\dot{z}_{j}(t- \zeta _{ij})\bigr) \\& \quad = 2\sum_{i=1}^{n}m_{i}^{2} \sum_{j=1}^{n}a_{ij} \mathcal{G}_{j}\bigl(z_{j}(t)\bigr) \sum _{k=1}^{n}e_{ik}\mathcal{F}_{k} \bigl(\dot{z}_{k}(t-\zeta _{ik})\bigr) \\& \quad = 2\sum_{i=1}^{n}\sum _{j=1}^{n}\sum_{k=1}^{n}m_{i}^{2}a_{ij}e_{ik} \mathcal{G}_{j}\bigl(z_{j}(t)\bigr) \mathcal{F}_{k}\bigl(\dot{z}_{k}(t-\zeta _{ik})\bigr) \\& \quad \leq 2\sum_{i=1}^{n} \sum_{j=1}^{n}\sum _{k=1}^{n}m_{i}^{2} \vert a_{ij} \vert \vert e_{ik} \vert \bigl\vert \mathcal{G}_{j}\bigl(z_{j}(t)\bigr) \bigr\vert \bigl\vert \mathcal{F}_{k}\bigl(\dot{z}_{k}(t-\zeta _{ik})\bigr) \bigr\vert \\& \quad \leq \sum_{i=1}^{n}\sum _{j=1}^{n}\sum_{k=1}^{n}m_{i}^{2} \vert a_{ij} \vert |e_{ik}| \bigl\vert \mathcal{G}_{j}^{2}\bigl(z_{j}(t)\bigr) \bigr\vert +\sum_{i=1}^{n}\sum _{j=1}^{n}\sum_{k=1}^{n}m_{i}^{2}|a_{ij}||e_{ik}| \mathcal{F}_{k}^{2}\bigl(\dot{z}_{k}(t- \zeta _{ik})\bigr) \\& \quad = \sum_{i=1}^{n}\sum _{j=1}^{n}\sum_{k=1}^{n}m_{i}^{2} \vert a_{ji} \vert |e_{jk}| \bigl\vert \mathcal{G}_{i}^{2}\bigl(z_{i}(t)\bigr) \bigr\vert +\sum_{i=1}^{n}\sum _{j=1}^{n}\sum_{k=1}^{n}m_{i}^{2}|a_{kj}||e_{ki}| \mathcal{F}_{i}^{2}\bigl(\dot{z}_{i}(t- \zeta _{ki})\bigr) \\& \quad \leq \sum_{i=1}^{n}\sum _{j=1}^{n}\sum_{k=1}^{n}m_{i}^{2} \vert a_{ji} \vert \vert e_{jk} \vert l_{i}^{2}z_{i}^{2}(t)+ \sum _{i=1}^{n}\sum _{j=1}^{n}\sum_{k=1}^{n}m_{i}^{4} \vert a_{jk} \vert \vert e_{ji} \vert \dot{z}_{i}^{2}(t-\zeta _{ji}) \\& \qquad (\text{according to $(P_{8, \text{new}})$--$(P_{9, \text{new}})$}). \end{aligned}$$

Thus, it yields

3.44 $$ \begin{aligned} &2\sum_{i=1}^{n}m_{i}^{2} \sum_{j=1}^{n}a_{ij} \mathcal{G}_{j}\bigl(z_{j}(t)\bigr) \sum _{j=1}^{n}e_{ij}\mathcal{F}_{j} \bigl(\dot{z}_{j}(t-\zeta _{ij})\bigr) \\ &\quad \leq \sum _{i=1}^{n}\sum_{j=1}^{n} \sum_{k=1}^{n}m_{i}^{2} \vert a_{ji} \vert \vert e_{jk} \vert l_{i}^{2}z_{i}^{2}(t)+ \sum _{i=1}^{n}\sum _{j=1}^{n}\sum_{k=1}^{n}m_{i}^{4} \vert a_{jk} \vert \vert e_{ji} \vert \dot{z}_{i}^{2}(t-\zeta _{ji}). \end{aligned} $$

Next, we focus on (3.36). So, we have the following:

$$\begin{aligned}& 2\sum_{i=1}^{n}m_{i}^{2} \sum_{j=1}^{n}b_{ij} \mathcal{G}_{j}\bigl(z_{j}(t-\tau _{ij}) \bigr)\sum_{j=1}^{n}e_{ij} \mathcal{F}_{j}\bigl( \dot{z}_{j}(t-\zeta _{ij})\bigr) \\& \quad = 2\sum_{i=1}^{n}m_{i}^{2} \sum_{j=1}^{n}b_{ij} \mathcal{G}_{j}\bigl(z_{j}(t- \tau _{ij}) \bigr)\sum_{k=1}^{n}e_{ik} \mathcal{F}_{k}\bigl(\dot{z}_{k}(t-\zeta _{ik})\bigr) \\& \quad = 2\sum_{i=1}^{n}\sum _{j=1}^{n}\sum_{k=1}^{n}m_{i}^{2}b_{ij}e_{ik} \mathcal{G}_{j}\bigl(z_{j}(t-\tau _{ij}) \bigr)\mathcal{F}_{k}\bigl(\dot{z}_{k}(t- \zeta _{ik})\bigr) \\& \quad \leq 2\sum_{i=1}^{n}\sum _{j=1}^{n}\sum_{k=1}^{n}m_{i}^{2} \vert b_{ij} \vert \vert e_{ik} \vert \bigl\vert \mathcal{G}_{j}\bigl(z_{j}(t-\tau _{ij})\bigr) \bigr\vert \bigl\vert \mathcal{F}_{k} \bigl(\dot{z}_{k}(t- \zeta _{ik})\bigr) \bigr\vert \\& \quad \leq \sum_{i=1}^{n}\sum _{j=1}^{n}\sum_{k=1}^{n}m_{i}^{2} \vert b_{ij} \vert \vert e_{ik} \vert \mathcal{G}_{j}^{2}\bigl(z_{j}(t-\tau _{ij})\bigr)+\sum_{i=1}^{n} \sum_{j=1}^{n} \sum _{k=1}^{n}m_{i}^{2} \vert b_{ij} \vert \vert e_{ik} \vert \mathcal{F}_{k}^{2}\bigl(\dot{z}_{k}(t- \zeta _{ik})\bigr) \\& \quad = \sum_{i=1}^{n}\sum _{j=1}^{n}\sum_{k=1}^{n}m_{i}^{2} \vert b_{ji} \vert \vert e_{jk} \vert \mathcal{G}_{i}^{2}\bigl(z_{i}(t-\tau _{ji})\bigr)+\sum_{i=1}^{n} \sum_{j=1}^{n} \sum _{k=1}^{n}m_{i}^{2} \vert b_{kj} \vert \vert e_{ki} \vert \mathcal{F}_{i}^{2}\bigl(\dot{z}_{i}(t- \zeta _{ki})\bigr) \\& \quad \leq \sum_{i=1}^{n}\sum _{j=1}^{n}\sum_{k=1}^{n}m_{i}^{2} \vert b_{ji} \vert \vert e_{jk} \vert l_{i}^{2}z_{i}^{2}(t- \tau _{ji})+\sum_{i=1}^{n}\sum _{j=1}^{n}\sum _{k=1}^{n}m_{i}^{4} \vert b_{jk} \vert \vert e_{ji} \vert \dot{z}_{i}^{2}(t-\zeta _{ji}) \\& \qquad (\text{based on $(P_{8, \text{new}})$--$(P_{9, \text{new}})$}). \end{aligned}$$

So, in a compact form, we get the following inequality:

3.45 $$ \begin{aligned} &2\sum_{i=1}^{n}m_{i}^{2} \sum_{j=1}^{n}b_{ij} \mathcal{G}_{j}\bigl(z_{j}(t-\tau _{ij}) \bigr)\sum_{j=1}^{n}e_{ij} \mathcal{F}_{j}\bigl( \dot{z}_{j}(t-\zeta _{ij})\bigr) \\ &\quad \leq \sum_{i=1}^{n}\sum _{j=1}^{n} \sum_{k=1}^{n}m_{i}^{2} \vert b_{ji} \vert \vert e_{jk} \vert l_{i}^{2}z_{i}^{2}(t-\tau _{ji})+ \sum_{i=1}^{n}\sum _{j=1}^{n}\sum _{k=1}^{n}m_{i}^{4} \vert b_{jk} \vert \vert e_{ji} \vert \dot{z}_{i}^{2}(t-\zeta _{ji}). \end{aligned} $$

Now we prepare ourselves to complete the mentioned unification process by estimation of the last triple series (3.37)–(3.39). To this aim, we first consider (3.37). Then, we have

$$ \begin{aligned} &\sum_{i=1}^{n}m_{i}^{2} \Biggl(\sum_{j=1}^{n}a_{ij} \mathcal{G}_{j}\bigl(z_{j}(t)\bigr) \Biggr) \Biggl(\sum _{j=1}^{n}a_{ij} \mathcal{G}_{j}\bigl(z_{j}(t)\bigr) \Biggr) \\ &\quad =\sum_{i=1}^{n}m_{i}^{2} \Biggl(\sum_{j=1}^{n}a_{ij} \mathcal{G}_{j}\bigl(z_{j}(t)\bigr) \Biggr) \Biggl(\sum _{k=1}^{n}a_{ij} \mathcal{G}_{j}\bigl(z_{j}(t)\bigr) \Biggr) \\ &\quad =\sum_{i=1}^{n}\sum _{j=1}^{n}\sum_{k=1}^{n}m_{i}^{2}a_{ij}a_{ik} \mathcal{G}_{j}\bigl(z_{j}(t)\bigr) \mathcal{G}_{k}\bigl(z_{k}(t)\bigr) \\ &\quad \leq \sum_{i=1}^{n}\sum _{j=1}^{n}\sum_{k=1}^{n}m_{i}^{2} \vert a_{ki} \vert \vert a_{kj} \vert \mathcal{G}_{i}^{2}\bigl(z_{i}(t)\bigr) \\ &\quad \leq \sum_{i=1}^{n}\sum _{j=1}^{n}\sum_{k=1}^{n}m_{i}^{2} \vert a_{ki} \vert \vert a_{kj} \vert l_{i}^{2}z_{i}^{2}(t)\quad (\text{based on the property $(P_{8, \text{new}})$}). \end{aligned} $$

Thus, it follows that

3.46 $$ \sum_{i=1}^{n}m_{i}^{2} \Biggl(\sum_{j=1}^{n}a_{ij} \mathcal{G}_{j}\bigl(z_{j}(t)\bigr) \Biggr) \Biggl(\sum _{j=1}^{n}a_{ij} \mathcal{G}_{j}\bigl(z_{j}(t)\bigr) \Biggr) \leq \sum _{i=1}^{n}\sum _{j=1}^{n}\sum_{k=1}^{n}m_{i}^{2} \vert a_{ki} \vert \vert a_{kj} \vert l_{i}^{2}z_{i}^{2}(t). $$

Prior to the final step, we have the triple (3.38) that gives us the following:

$$\begin{aligned}& \sum_{i=1}^{n}m_{i}^{2} \Biggl(\sum_{j=1}^{n}b_{ij} \mathcal{G}_{j}\bigl(z_{j}(t-\tau _{ij}) \bigr) \Biggr) \Biggl(\sum_{j=1}^{n}b_{ij} \mathcal{G}_{j}\bigl(z_{j}(t-\tau _{ij}) \bigr) \Biggr) \\& \quad = \sum_{i=1}^{n}m_{i}^{2} \Biggl(\sum_{j=1}^{n}b_{ij} \mathcal{G}_{j}\bigl(z_{j}(t- \tau _{ij}) \bigr) \Biggr) \Biggl(\sum_{k=1}^{n}b_{ik} \mathcal{G}_{k}\bigl(z_{k}(t- \tau _{ik}) \bigr) \Biggr) \\& \quad = \sum_{i=1}^{n}\sum _{j=1}^{n}\sum_{k=1}^{n}m_{i}^{2}b_{ij}b_{ik} \mathcal{G}_{j}\bigl(z_{j}(t-\tau _{ij}) \bigr)\mathcal{G}_{k}\bigl(z_{k}(t-\tau _{ik})\bigr) \\& \quad \leq \sum_{i=1}^{n}\sum _{j=1}^{n}\sum_{k=1}^{n}m_{i}^{2} \vert b_{ij} \vert \vert b_{ik} \vert \bigl\vert \mathcal{G}_{j}\bigl(z_{j}(t-\tau _{ij})\bigr) \bigr\vert \bigl\vert \mathcal{G}_{k} \bigl(z_{k}(t-\tau _{ik})\bigr) \bigr\vert \\& \quad \leq \frac{1}{2}\sum_{i=1}^{n} \sum_{j=1}^{n}\sum _{k=1}^{n}m_{i}^{2} \vert b_{ij} \vert \vert b_{ik} \vert \mathcal{G}_{j}^{2}\bigl(z_{j}(t-\tau _{ij})\bigr)+\frac{1}{2}\sum_{i=1}^{n} \sum_{j=1}^{n}\sum _{k=1}^{n}m_{i}^{2} \vert b_{ij} \vert \vert b_{ik} \vert \mathcal{G}_{k}^{2}\bigl(z_{k}(t- \tau _{ik})\bigr) \\& \quad = \frac{1}{2}\sum_{i=1}^{n} \sum_{j=1}^{n}\sum _{k=1}^{n}m_{i}^{2} \vert b_{ji} \vert \vert b_{jk} \vert \mathcal{G}_{i}^{2}\bigl(z_{i}(t-\tau _{ji})\bigr)+\frac{1}{2}\sum_{i=1}^{n} \sum_{j=1}^{n}\sum _{k=1}^{n}m_{i}^{2} \vert b_{kj} \vert \vert b_{ki} \vert \mathcal{G}_{i}^{2}\bigl(z_{i}(t- \tau _{ki})\bigr) \\& \quad = \frac{1}{2}\sum _{i=1}^{n}\sum_{j=1}^{n} \sum_{k=1}^{n}m_{i}^{2} \vert b_{ji} \vert \vert b_{jk} \vert \mathcal{G}_{i}^{2}\bigl(z_{i}(t-\tau _{ji})\bigr)+\frac{1}{2}\sum_{i=1}^{n} \sum_{j=1}^{n}\sum _{k=1}^{n}m_{i}^{2} \vert b_{jk} \vert \vert b_{ji} \vert \mathcal{G}_{i}^{2}\bigl(z_{i}(t- \tau _{ji})\bigr) \\& \quad = \sum_{i=1}^{n}\sum _{j=1}^{n}\sum_{k=1}^{n}m_{i}^{2} \vert b_{ji} \vert \vert b_{jk} \vert \mathcal{G}_{i}^{2}\bigl(z_{i}(t-\tau _{ji})\bigr) \\& \quad \leq \sum_{i=1}^{n}\sum _{j=1}^{n}\sum_{k=1}^{n}m_{i}^{2} \vert b_{ji} \vert \vert b_{jk} \vert l_{i}^{2}z_{i}^{2}(t- \tau _{ji})\quad (\text{based on the property $(P_{8, \text{new}})$}). \end{aligned}$$

Equivalently, it has demonstrated that

3.47 $$ \begin{aligned} &\sum_{i=1}^{n}m_{i}^{2} \Biggl(\sum_{j=1}^{n}b_{ij} \mathcal{G}_{j}\bigl(z_{j}(t- \tau _{ij}) \bigr) \Biggr) \Biggl(\sum_{j=1}^{n}b_{ij} \mathcal{G}_{j}\bigl(z_{j}(t- \tau _{ij}) \bigr) \Biggr) \\ &\quad \leq \sum_{i=1}^{n}\sum _{j=1}^{n}\sum _{k=1}^{n}m_{i}^{2} \vert b_{ji} \vert \vert b_{jk} \vert l_{i}^{2}z_{i}^{2}(t- \tau _{ji}). \end{aligned} $$

This is the final step, where we estimate the triple series (3.39). Hence, one has

$$\begin{aligned}& \sum_{i=1}^{n}m_{i}^{2} \Biggl(\sum_{j=1}^{n}e_{ij} \mathcal{F}_{j}\bigl(\dot{z}_{j}(t-\zeta _{ij})\bigr) \Biggr) \Biggl(\sum_{j=1}^{n}e_{ij} \mathcal{F}_{j}\bigl(\dot{z}_{j}(t-\zeta _{ij})\bigr) \Biggr) \\& \quad = \sum_{i=1}^{n}m_{i}^{2} \Biggl(\sum_{j=1}^{n}e_{ij} \mathcal{F}_{j}\bigl( \dot{z}_{j}(t-\zeta _{ij})\bigr) \Biggr) \Biggl(\sum_{k=1}^{n}e_{ik} \mathcal{F}_{k}\bigl(\dot{z}_{k}(t-\zeta _{ik})\bigr) \Biggr) \\& \quad = \sum_{i=1}^{n}\sum _{j=1}^{n}\sum_{k=1}^{n}m_{i}^{2}e_{ij}e_{ik} \mathcal{F}_{j}\bigl(\dot{z}_{j}(t-\zeta _{ij})\bigr)\mathcal{F}_{k}\bigl(\dot{z}_{k}(t- \zeta _{ik})\bigr) \\& \quad \leq \sum_{i=1}^{n}\sum _{j=1}^{n}\sum_{k=1}^{n}m_{i}^{2} \vert e_{ij} \vert \vert e_{ik} \vert \bigl\vert \mathcal{F}_{j}\bigl(\dot{z}_{j}(t-\zeta _{ij})\bigr) \bigr\vert \bigl\vert \mathcal{F}_{k} \bigl(z_{k}(t- \zeta _{ik})\bigr) \bigr\vert \\& \quad \leq \frac{1}{2}\sum_{i=1}^{n} \sum_{j=1}^{n}\sum _{k=1}^{n}m_{i}^{2} \vert e_{ij} \vert \vert e_{ik} \vert \mathcal{F}_{j}^{2}\bigl(\dot{z}_{j}(t-\zeta _{ij})\bigr)+\frac{1}{2}\sum_{i=1}^{n} \sum_{j=1}^{n}\sum _{k=1}^{n}m_{i}^{2} \vert e_{ij} \vert \vert e_{ik} \vert \mathcal{F}_{k}^{2}\bigl( \dot{z}_{k}(t- \zeta _{ik})\bigr) \\& \quad = \frac{1}{2}\sum_{i=1}^{n} \sum_{j=1}^{n}\sum _{k=1}^{n}m_{i}^{2} \vert e_{ji} \vert \vert e_{jk} \vert \mathcal{F}_{i}^{2}\bigl(\dot{z}_{i}(t-\zeta _{ji})\bigr)+\frac{1}{2}\sum_{i=1}^{n} \sum_{j=1}^{n}\sum _{k=1}^{n}m_{i}^{2} \vert e_{kj} \vert \vert e_{ki} \vert \mathcal{F}_{i}^{2}\bigl( \dot{z}_{i}(t- \zeta _{ki})\bigr) \\& \quad = \frac{1}{2}\sum_{i=1}^{n} \sum_{j=1}^{n}\sum _{k=1}^{n}m_{i}^{2} \vert e_{ji} \vert \vert e_{jk} \vert \mathcal{F}_{i}^{2}\bigl(\dot{z}_{i}(t-\zeta _{ji})\bigr)+\frac{1}{2}\sum_{i=1}^{n} \sum_{j=1}^{n}\sum _{k=1}^{n}m_{i}^{2} \vert e_{jk} \vert \vert e_{ji} \vert \mathcal{F}_{i}^{2}\bigl( \dot{z}_{i}(t- \zeta _{ji})\bigr) \\& \quad = \sum_{i=1}^{n}\sum _{j=1}^{n}\sum_{k=1}^{n}m_{i}^{2} \vert e_{ji} \vert \vert e_{jk} \vert \mathcal{F}_{i}^{2}\bigl(\dot{z}_{i}(t-\zeta _{ji})\bigr) \\& \quad \leq \sum_{i=1}^{n}\sum _{j=1}^{n}\sum_{k=1}^{n}m_{i}^{4} \vert e_{ji} \vert \vert e_{jk} \vert \dot{z}_{i}^{2}(t-\zeta _{ji})\quad (\text{based on the property $(P_{9, \text{new}})$}). \end{aligned}$$

In a compact form, we have proven that

3.48 $$ \begin{aligned} &\sum_{i=1}^{n}m_{i}^{2} \Biggl(\sum_{j=1}^{n}e_{ij} \mathcal{F}_{j}\bigl( \dot{z}_{j}(t-\zeta _{ij})\bigr) \Biggr) \Biggl(\sum_{j=1}^{n}e_{ij} \mathcal{F}_{j}\bigl(\dot{z}_{j}(t-\zeta _{ij})\bigr) \Biggr) \\ &\quad \leq \sum_{i=1}^{n} \sum_{j=1}^{n}\sum _{k=1}^{n}m_{i}^{4} \vert e_{ji} \vert \vert e_{jk} \vert \dot{z}_{i}^{2}(t- \zeta _{ji}). \end{aligned} $$

Now, let us compare (3.31)–(3.39) with (3.40)–(3.48) and then gather the obtained data into (3.27). In this case, we come to the conclusion that

3.49 $$ \begin{aligned} &\dot{V}_{1}\bigl(z(t), \dot{z}(t),t\bigr) \\ &\quad :=(1+\alpha )\sum_{i=1}^{n}c_{i}m_{i}^{2}z_{i}(t) \dot{z}_{i}(t)+\frac{1}{n}\sum _{i=1}^{n}\sum_{j=1}^{n} \mathcal{F}_{j}^{2}\bigl( \dot{z}_{j}(t) \bigr)-\frac{1}{n}\sum_{i=1}^{n} \sum_{j=1}^{n}\mathcal{F}_{j}^{2} \bigl( \dot{z}_{j}(t-\zeta _{ij})\bigr) \\ &\quad \leq -\alpha \sum_{i=1}^{n}m_{i}^{2}c_{i}^{2}z_{i}^{2}(t) \\ &\qquad {}+\frac{1-\alpha }{2}\sum_{i=1}^{n} \sum_{j=1}^{n}m_{i}^{2}c_{i} \bigl( \vert a_{ij} \vert +l_{i}^{2} \vert a_{ji} \vert \bigr)z_{i}^{2}(t) \\ &\qquad {}+\frac{1-\alpha }{2}\sum_{i=1}^{n} \sum_{j=1}^{n}m_{i}^{2}c_{i} \vert b_{ij} \vert z_{i}^{2}(t)+ \frac{1-\alpha }{2}\sum_{i=1}^{n}\sum _{j=1}^{n}m_{i}^{2}c_{i} \vert b_{ji} \vert l_{i}^{2}z_{i}^{2}(t- \tau _{ji}) \\ &\qquad {}+\frac{1-\alpha }{2}\sum_{i=1}^{n} \sum_{j=1}^{n}m_{i}^{2}c_{i} \vert e_{ij} \vert z_{i}^{2}(t)+ \frac{1-\alpha }{2}\sum_{i=1}^{n}\sum _{j=1}^{n}m_{i}^{4}c_{i} \vert e_{ji} \vert \dot{z}_{i}^{2}(t- \zeta _{ji}) \\ &\qquad {}+\sum_{i=1}^{n}\sum _{j=1}^{n}\sum_{k=1}^{n}m_{i}^{2} \vert a_{ji} \vert \vert b_{jk} \vert l_{i}^{2}z_{i}^{2}(t)+ \sum _{i=1}^{n}\sum _{j=1}^{n}\sum_{k=1}^{n}m_{i}^{2} \vert a_{jk} \vert \vert b_{ji} \vert l_{i}^{2}z_{i}^{2}(t- \tau _{ji}) \\ &\qquad {}+\sum_{i=1}^{n}\sum _{j=1}^{n}\sum_{k=1}^{n}m_{i}^{2} \vert a_{ji} \vert \vert e_{jk} \vert l_{i}^{2}z_{i}^{2}(t)+ \sum _{i=1}^{n}\sum _{j=1}^{n}\sum_{k=1}^{n}m_{i}^{4} \vert a_{jk} \vert \vert e_{ji} \vert \dot{z}_{i}^{2}(t-\zeta _{ji}) \\ &\qquad {}+\sum_{i=1}^{n}\sum _{j=1}^{n}\sum_{k=1}^{n}m_{i}^{2} \vert b_{ji} \vert \vert e_{jk} \vert l_{i}^{2}z_{i}^{2}(t- \tau _{ji})+\sum_{i=1}^{n}\sum _{j=1}^{n}\sum _{k=1}^{n}m_{i}^{4} \vert b_{jk} \vert \vert e_{ji} \vert \dot{z}_{i}^{2}(t-\zeta _{ji}) \\ &\qquad {}+\sum_{i=1}^{n}\sum _{j=1}^{n}\sum_{k=1}^{n}m_{i}^{2} \vert a_{ki} \vert \vert a_{kj} \vert l_{i}^{2}z_{i}(t) \\ &\qquad {}+\sum_{i=1}^{n}\sum _{j=1}^{n}\sum_{k=1}^{n}m_{i}^{2} \vert b_{ji} \vert \vert b_{jk} \vert l_{i}^{2}z_{i}^{2}(t- \tau _{ji}) \\ &\qquad {}+\sum_{i=1}^{n}\sum _{j=1}^{n}\sum_{k=1}^{n}m_{i}^{4} \vert e_{ji} \vert \vert e_{jk} \vert \dot{z}_{i}^{2}(t-\zeta _{ji}). \end{aligned} $$

Since

3.50 $$ \dot{V}\bigl(z(t),\dot{z}(t),t\bigr):=\dot{V}_{1} \bigl(z(t),\dot{z}(t),t\bigr)+\dot{V}_{2}\bigl(z(t), \dot{z}(t),t \bigr), $$

then, in the light of (3.27), (3.28), and some simplifications, we arrive at

3.51 $$ \begin{aligned} &\dot{V}\bigl(z(t),\dot{z}(t),t\bigr) \\ &\quad \leq \sum_{i=1}^{n}m_{i}^{2} \Biggl\{ -\alpha \bigl(c_{i}^{2}+\delta l_{i}^{2} \bigr)+ \frac{1-\alpha }{2}\sum _{j=1}^{n}\bigl\{ \vert a_{ij} \vert + \vert b_{ij} \vert + \vert e_{ij} \vert +l_{i}^{2} \vert a_{ji} \vert \bigr\} \\ &\qquad {} + l_{i}^{2}\sum_{j=1}^{n} \sum_{k=1}^{n}\bigl\{ \vert a_{ji} \vert \vert b_{jk} \vert + \vert a_{ji} \vert \vert c_{jk} \vert + \vert a_{ki} \vert \vert a_{kj} \vert \bigr\} \Biggr\} z_{i}^{2}(t) \\ &\qquad {}+\sum_{i=1}^{n}l_{i}^{2} \Biggl\{ c_{i}\frac{1-\alpha }{2}\sum_{j=1}^{n} \vert b_{ji} \vert - \sum_{j=1}^{n} \sum_{k=1}^{n}\bigl\{ \vert a_{jk} \vert \vert b_{ji} \vert + \vert b_{jk} \vert \vert b_{ji} \vert + \vert e_{jk} \vert \vert c_{ji} \vert \bigr\} \Biggr\} \\ &\qquad {}\times z_{i}^{2}(t-\tau _{ji}) \\ &\qquad {}+\sum_{i=1}^{n}m_{i}^{4} \Biggl\{ c_{i}\frac{1-\alpha }{2}\sum_{j=1}^{n} \vert e_{ji} \vert - \sum_{j=1}^{n} \sum_{k=1}^{n}\bigl\{ \vert a_{jk} \vert \vert e_{ji} \vert + \vert b_{jk} \vert \vert e_{ji} \vert + \vert e_{jk} \vert \vert e_{ji} \vert \bigr\} \Biggr\} \\ &\qquad {}\times \dot{z}_{i}^{2}(t-\zeta _{ji}) \\ &\quad =-\sum_{i=1}^{n}m_{i}^{2} \epsilon _{1,i}z_{i}^{2}(t)-\sum _{i=1}^{n}l_{i}^{2} \epsilon _{2,i}z_{i}^{2}(t-\tau _{ji})-\sum_{i=1}^{n}m_{i}^{4} \epsilon _{3,i}\dot{z}_{i}^{2}(t-\zeta _{ji}). \end{aligned} $$

Since, for each $i=1,2,\dots ,n$, the constants $m_{i}^{2}$, $l_{i}^{2}$, $\epsilon _{1,i}$, $\epsilon _{2,i}$, and $\epsilon _{3,i}$ all are positive, this is a direct consequence of the inequality (3.51), that is, $\dot{V}(z(t),\dot{z}(t),t)<0$, except that for each $i,j=1,2,\dots ,n$, $z_{i}(t):=0$, $z_{i}(t-\tau _{ij}):=0$, and $\dot{z}_{i}(t-\zeta _{ij}):=0$, that is, the origin $z(t)=[z_{1}(t),z_{2}(t),\dots ,z_{n}(t)]^{T}=[0,0,\dots ,0]^{T}$ is asymptotically stable. Besides, if $\|z(t)\|_{Y}\to \infty $, then, according to (3.24) and (3.25), $\dot{V}(z(t),\dot{z}(t),t)\to \infty $, that is, the Lyapunov functional (3.24)–(3.26) is radially unbounded. So, the origin $z(t):=0$ is globally asymptotically stable. This completes the stability analysis of the NNTDNN (3.17). □

## Numerical simulations

This section is devoted to the numerical applications illustrating the implementability of the stability criterion presented in Theorem 3.2.

### Example 4.1

Let us consider the NNTDNN

4.1 $$ \textstyle\begin{cases} \dot{x}(t):=-x(t)+2\sin (x(t))-\frac{1}{2}\sin (x(t-0.1))- \frac{1}{2}\sin (x(t-5)) \\ \hphantom{\dot{x}(t):={}}{}-\frac{1}{2}\dot{x}(t-0.1)-\frac{1}{2} \dot{x}(t-0.4), \\ \dot{y}(t):=-y(t)+2\sin (y(t))-\frac{1}{2}\sin (x(t-3))-\frac{1}{2} \sin (x(t-1)) \\ \hphantom{\dot{y}(t):={}}{}-\frac{1}{2}\dot{y}(t-1)-\frac{1}{2}\dot{y}(t-0.4). \end{cases} $$

Indeed, the neural dynamical system (4.1) is a reduced version of the NNTDNN (3.17) for $n=2$, having the following coefficient matrices:

4.2 $$\begin{array}{rl} & C:=\left[\begin{array}{c} c\\ c \end{array}\right],\;A:=\left[\begin{array}{cc} a & a\\ a & a \end{array}\right],\;B:=\left[\begin{array}{cc} b & b\\ b & b \end{array}\right],\\ & E:=\left[\begin{array}{cc} e & e\\ e & e \end{array}\right],\;c=a:=1,\;b=e:=-\frac{1}{2}. \end{array}$$

Furthermore, the time delays and activation functions have been chosen as follows:

4.3 $$ \begin{aligned} &\textstyle\begin{cases} \tau _{11}:=0.1,\qquad \tau _{12}:=5, \qquad \tau _{21}:=3, \qquad \tau _{22}:=1, \\ \zeta _{11}:=0.1, \qquad \zeta _{12}:=0.4, \qquad \zeta _{21}:=1, \qquad \zeta _{22}:=0.4, \end{cases}\displaystyle \quad \text{and} \\ &\textstyle\begin{cases} \mathcal{G}_{i}(w):=\sin (w), \quad i=1,2, \\ \mathcal{F}_{i}(w):=w, \quad i=1,2. \end{cases}\displaystyle \end{aligned} $$

In this case, $l_{i}=m_{i}:=1$, $i=1,2$, and $\delta :=5$. Having the above mentioned data in hand, it is easy to check that, for $\alpha =0.25$ and each $i=1,2$,

4.4 $$ \epsilon _{1,i}>0, \qquad \epsilon _{2,i}>0,\qquad \epsilon _{3,i}>0. $$

**Figure 1:**
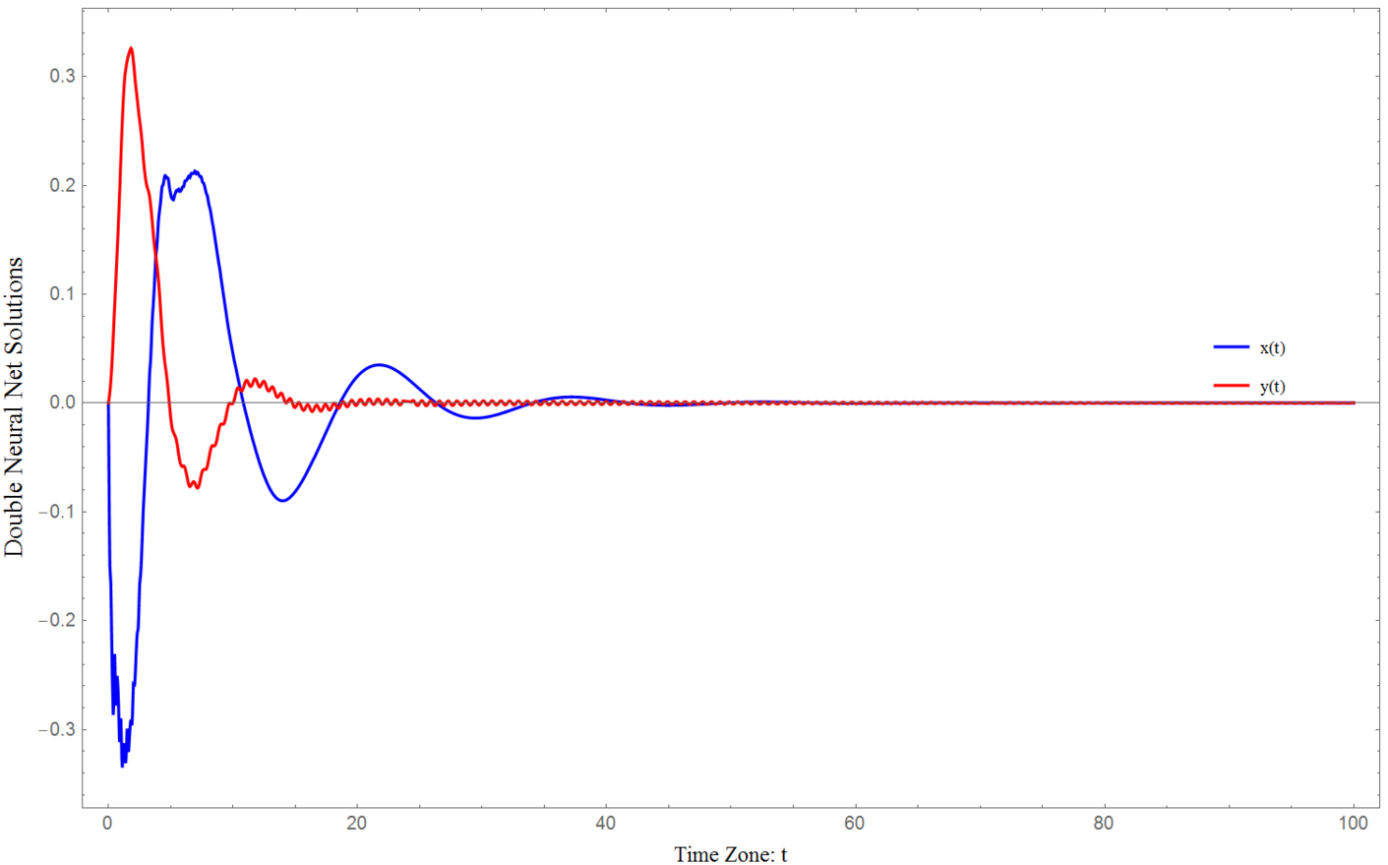
Global asymptotical stability of the origin in the NNTDNN (4.1)

Since all conditions of Theorem 3.2 are satisfied, we come to the conclusion that the origin of the NNTDNN (4.1) is globally asymptotically stable, as can be observed in the following numerical simulation (see Fig. 1).

### Example 4.2

In this simulation we choose $n=4$ in the NNTDNN (3.17) and consider the following neural network:

4.5 $$ \textstyle\begin{cases} \dot{x}(t):=-x(t)-\sin (x(t-0.1))-\sin (x(t-0.2))-\sin (x(t-0.3))- \sin (x(t-5)) \\ \hphantom{\dot{x}(t):={}}{} -\frac{1}{16}\dot{x}(t-0.1)-\frac{1}{16}\dot{x}(t-0.02)- \frac{1}{16}\dot{x}(t-0.03)-\frac{1}{16}\dot{x}(t-0.4), \\ \dot{y}(t):=-y(t)-\sin (2x(t-0.1))-\sin (2y(t-0.2))-\sin (2y(t-0.3)) \\ \hphantom{\dot{y}(t):={}}{} - \sin (2y(t-4))-\frac{1}{16}\dot{y}(t-0.1)-\frac{1}{16}\dot{y}(t-0.02)- \frac{1}{16}\dot{y}(t-0.03) \\ \hphantom{\dot{y}(t):={}}{}-\frac{1}{16}\dot{y}(t-0.4), \\ \dot{z}(t):=-z(t)-\sin (4z(t-0.3))-\sin (4z(t-0.04))-\sin (4z(t-0.05)) \\ \hphantom{\dot{z}(t):={}}{} - \sin (4z(t-1))-\frac{1}{16}\dot{z}(t-0.1)-\frac{1}{16}\dot{z}(t-0.02)- \frac{1}{16}\dot{z}(t-0.03) \\ \hphantom{\dot{z}(t):={}}{}-\frac{1}{16}\dot{z}(t-0.4), \\ \dot{w}(t):=-w(t)-\sin (w(t-0.4))-\sin (w(t-0.6))-\sin (w(t-0.8)) \\ \hphantom{\dot{w}(t):={}}{} - \sin (w(t-2))-\frac{1}{16}\dot{w}(t-0.1)-\frac{1}{16}\dot{w}(t-0.02)- \frac{1}{16}\dot{w}(t-0.03) \\ \hphantom{\dot{w}(t):={}}{}-\frac{1}{16}\dot{w}(t-0.4). \end{cases} $$

In this case, we can summarize the coefficients of the neural network (4.5) as follows:

4.6 $$\begin{array}{rl} & C:=\left[\begin{array}{c} c\\ c\\ c\\ c \end{array}\right],\;A:=\left[\begin{array}{cccc} a & a & a & a\\ a & a & a & a\\ a & a & a & a\\ a & a & a & a \end{array}\right],\;B:=-\left[\begin{array}{cccc} b & b & b & b\\ b & b & b & b\\ b & b & b & b\\ b & b & b & b \end{array}\right],\\ & E:=-\frac{1}{16}\left[\begin{array}{cccc} e & e & e & e\\ e & e & e & e\\ e & e & e & e\\ e & e & e & e \end{array}\right],\;c=b=e:=1,\;a:=0. \end{array}$$

Besides, the time delays of the NNTDNN (4.6) are as follows:

4.7 $$ \textstyle\begin{cases} \tau _{11}:=0.1, \qquad \tau _{12}:=0.2, \qquad \tau _{13}:=0.3,\qquad \tau _{14}:=5, \\ \tau _{21}:=0.1, \qquad \tau _{22}:=0.2, \qquad \tau _{23}:=0.3,\qquad \tau _{24}:=4, \\ \tau _{31}:=0.3, \qquad \tau _{32}:=0.04,\qquad \tau _{33}:=0.05,\qquad \tau _{34}:=1, \\ \tau _{41}:=0.4,\qquad \tau _{42}:=0.6,\qquad \tau _{43}:=0.8,\qquad \tau _{44}:=2, \\ \zeta _{11}:=0.1,\qquad \zeta _{12}:=0.02,\qquad \zeta _{13}:=0.03,\qquad \zeta _{14}:=0.4, \\ \zeta _{21}:=0.1, \qquad \zeta _{22}:=0.02,\qquad \zeta _{23}:=0.03,\qquad \zeta _{24}:=0.4, \\ \zeta _{31}:=0.1,\qquad \zeta _{32}:=0.02,\qquad \zeta _{33}:=0.03,\qquad \zeta _{34}:=0.4, \\ \zeta _{41}:=0.1, \qquad \zeta _{42}:=0.02,\qquad \zeta _{43}:=0.3,\qquad \zeta _{44}:=0.04. \end{cases} $$

So, we get that $\delta :=5$. Finally, according to the neural dynamical system (4.5), we have

4.8 $$ \textstyle\begin{cases} \mathcal{G}_{1}(z)=\mathcal{G}_{4}(z):=\sin (z)\Longrightarrow l_{1}=l_{4}:=1, \\ \mathcal{G}_{2}(z):=\sin (2z)\Longrightarrow l_{2}:=2, \\ \mathcal{G}_{3}(z):=\sin (4z)\Longrightarrow l_{3}:=3, \\ \mathcal{F}_{i}(v):=v\Longrightarrow m_{i}:=1, \quad i=1,2,3,4. \end{cases} $$

Keeping the aforementioned setting and choosing $\alpha =0.5$, with a direct computation, it is concluded that

**Figure 2:**
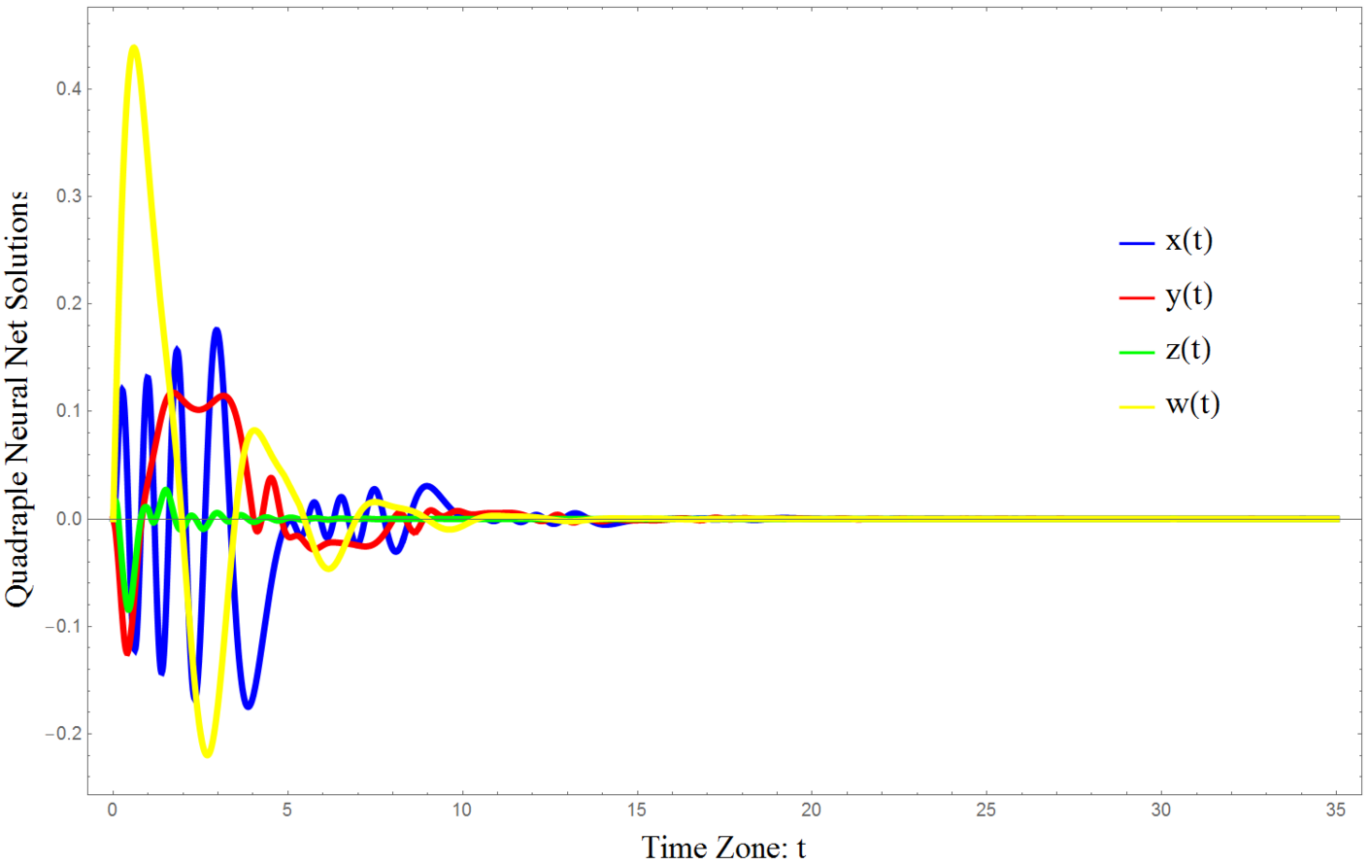
Global asymptotical stability of the origin in the NNTDNN (4.5)

4.9 $$ \epsilon _{1,i}, \epsilon _{2,i}, \epsilon _{3,i}, \epsilon _{4,i}>0, \quad i=1,2,3,4, $$

that is, the origin of the NNTDNN (4.5) is globally asymptotically stable, as is shown in Fig. 2.

## Concluding remarks

We finalize this paper with a compact description of the managed investigation. In this paper, the nonlinear neutral-type time-delayed neural network (2.1) has been studied. The main aim of our investigation was to introduce a novel Lyapunov functional to stabilize this neural dynamical system. Since the NNTDNN (2.1) cannot be represented in the vector matrix form, such as the matrix neural network (2.7), it cannot be stabilized with some standard stability tools such as the linear matrix inequalities technique. Therefore, the quadratic-integral Lyapunov functional (3.24)–(3.26) has been introduced as the basic stability key for the global asymptotical stabilization of the NNTDNN (2.1). In what follows, we summarize the steps of our investigation:

$(S_{1})$
In this paper, we consider a mathematical model of the *n* interconnecting neurons (2.1).
$(S_{2})$
This neural network model is nonlinear, that makes it more suitable for studying the real world phenomena.
$(S_{3})$
This neuro-system is of neutral type, meaning that not only the neuron states but also the states of their derivatives appear in the nonlinearities which makes it a more accurate mathematical statement of the studied model.
$(S_{4})$
This model includes multiple time delays, which have important roles in the (dis)appearance of the stability and oscillation within the model (as illustrated in the numerical simulations).
$(S_{5})$
In this paper, we apply the coincidence degree theory to solve the neural dynamical system (2.1), which is a rare mathematical solvability tool for the neural networks.
$(S_{6})$
After guaranteeing the existence of at least one solution for the NNTDNN (2.1), in order to reach a unique solution, we have transformed the neuro-system (2.1) to the NNTDNN (3.17) that has the origin as its unique solution.
$(S_{7})$
In this paper, we have defined the new quadratic-integral Lyapunov functional (3.24)-(3.26) including all of the time delays to globally asymptotically stabilization of the transformed neuro-system (3.17).
$(S_{8})$
At the end of the stability analysis, some numerical simulations have been presented, which justify the implementability of the presented stability criterion.

## Data Availability

Not applicable.
